# Using iterative cluster merging with improved gap statistics to perform online phenotype discovery in the context of high-throughput RNAi screens

**DOI:** 10.1186/1471-2105-9-264

**Published:** 2008-06-05

**Authors:** Zheng Yin, Xiaobo Zhou, Chris Bakal, Fuhai Li, Youxian Sun, Norbert Perrimon, Stephen TC Wong

**Affiliations:** 1 Center for Bioinformatics, The Methodist Hospital Research Institute and Weill Cornell College of Medicine, 6565 Fannin Street, Houston, TX, 77030, USA; 2 Department of Genetics and Howard Hughes Medical Institute, Harvard Medical School, 77 Avenue Louis Pasteur, Boston, MA, 02115, USA; 3 State Key Laboratory of Industrial Control Technology, Zhejiang University, 38 Zheda Road, Hangzhou, Zhejiang Province, 310027, PR China

## Abstract

**Background:**

The recent emergence of high-throughput automated image acquisition technologies has forever changed how cell biologists collect and analyze data. Historically, the interpretation of cellular phenotypes in different experimental conditions has been dependent upon the expert opinions of well-trained biologists. Such qualitative analysis is particularly effective in detecting subtle, but important, deviations in phenotypes. However, while the rapid and continuing development of automated microscope-based technologies now facilitates the acquisition of trillions of cells in thousands of diverse experimental conditions, such as in the context of RNA interference (RNAi) or small-molecule screens, the massive size of these datasets precludes human analysis. Thus, the development of automated methods which aim to identify novel and biological relevant phenotypes online is one of the major challenges in high-throughput image-based screening. Ideally, phenotype discovery methods should be designed to utilize prior/existing information and tackle three challenging tasks, i.e. restoring pre-defined biological meaningful phenotypes, differentiating novel phenotypes from known ones and clarifying novel phenotypes from each other. Arbitrarily extracted information causes biased analysis, while combining the complete existing datasets with each new image is intractable in high-throughput screens.

**Results:**

Here we present the design and implementation of a novel and robust online phenotype discovery method with broad applicability that can be used in diverse experimental contexts, especially high-throughput RNAi screens. This method features phenotype modelling and iterative cluster merging using improved gap statistics. A Gaussian Mixture Model (GMM) is employed to estimate the distribution of each existing phenotype, and then used as reference distribution in gap statistics. This method is broadly applicable to a number of different types of image-based datasets derived from a wide spectrum of experimental conditions and is suitable to adaptively process new images which are continuously added to existing datasets. Validations were carried out on different dataset, including published RNAi screening using *Drosophila *embryos [Additional files 1, 2], dataset for cell cycle phase identification using HeLa cells [Additional files 1, 3, 4] and synthetic dataset using polygons, our methods tackled three aforementioned tasks effectively with an accuracy range of 85%–90%. When our method is implemented in the context of a *Drosophila *genome-scale RNAi image-based screening of cultured cells aimed to identifying the contribution of individual genes towards the regulation of cell-shape, it efficiently discovers meaningful new phenotypes and provides novel biological insight. We also propose a two-step procedure to modify the novelty detection method based on one-class SVM, so that it can be used to online phenotype discovery. In different conditions, we compared the SVM based method with our method using various datasets and our methods consistently outperformed SVM based method in at least two of three tasks by 2% to 5%. These results demonstrate that our methods can be used to better identify novel phenotypes in image-based datasets from a wide range of conditions and organisms.

**Conclusion:**

We demonstrate that our method can detect various novel phenotypes effectively in complex datasets. Experiment results also validate that our method performs consistently under different order of image input, variation of starting conditions including the number and composition of existing phenotypes, and dataset from different screens. In our findings, the proposed method is suitable for online phenotype discovery in diverse high-throughput image-based genetic and chemical screens.

## Background

Metazoan cells have the ability to adopt an extraordinarily diverse spectrum of cell shapes. For example, the cuboidal, polarized morphology of epithelial cells differs markedly from that of neuronal cells, which extend long, thin, and highly-branched projections. The shape of an individual cell is the result of a complex interplay between the activity of thousands of genes and the cell's environment. Understanding this interplay is a fundamental challenge in developmental and cell biology. Currently, there are two key aspects to deciphering cellular morphogenesis on genome-scale. The first is determining the individual functional contributions of every gene towards the regulation of cell shape, and the second is to describe how complex relationships between cell shape genes affect morphology. With the advent of high-throughput RNA interference (RNAi) screening technologies, particularly in model systems such as *Drosophila melanogaster *[1], it is now possible to systematically query the involvement of genes in the regulation of different cellular processes and functions. Typically, RNAi-based genetic screens involve the acquisition of relatively low-content, single-dimensional data which is easily analyzed using conventional and unbiased means and thus feasible to perform on genome, or multi-genome scales [1,2]. In order to facilitate similar analysis of image-based screens, we and other researchers have recently developed novel image segmentation algorithms to rapidly quantitate hundreds of different parameters at a single-cell level in an automated fashion [3-6], and we have demonstrated that such image segmentation algorithms can be used in the context of genetic screens [7]. Notably however, this and other similar screens [8] have been 50–100 fold smaller in scale than typical low-dimensional screens and are not yet genome-scale. The reduced scale of these screens is due, largely in part, to the fact that the expert opinion of cell biologists is still an essential and rate-limiting aspect in the analysis of many image-based datasets. Although human intervention is not required in screens where the potential phenotypic outcomes are few or binary in number (e.g. an image-based screen where a particular marker is determined to be nuclear or non-nuclear), such intervention is currently necessary in order to identify novel/subtle phenotypes in image-based datasets of genetic or chemical perturbations where the dynamic range of cellular phenotypes cannot be predicted before the data is collected. For example, in genome-scale screens for regulators of cell shape, it is impossible to predict *a priori *the diversity of morphologies that will ultimately be present in the dataset. The failure to accurately measure this phenotypic variation will lead to concomitant classification errors, especially false negatives, and misleading results. Current methodologies usually employ a two-step procedure to maximize the amount of variation that is captured in a particular image-based analysis. *First*, 100–600 phenotypic features are measured on a single-cell level (automatically but somehow exhaustively), and *second*, supervised techniques assisted by biologists are used to both reduce dimensionality of feature space and carrying out classification on the images. The biologist has to at least perform preliminary qualitative visual scoring of a small part of the dataset in order to gain a crude assessment of the phenotypic variance that is present in this subset. Unfortunately, it is impossible to perform such analysis in the course of screens where millions of images are acquired, thus the ability of these screens to identify new phenotypes is greatly limited. The issues of defining meaningful phenotypes and describing them using informative feature subsets are closely related. Automated feature space reduction schemes have been implemented in the context of high content screen, including feature extraction methods examined in [9], factor analysis in [10] and SVM-RFE method in [11]. These methods allow more effective modelling of existing phenotypes, and also prompt the necessity of updating informative feature sets so that they can not only model the existing, but also discover the novel.

Cluster analysis is widely used to reveal the structure of unlabeled datasets. Specifically, there are a number of methods that have been developed in order to estimate cluster numbers from a dataset such as using a series of internal indices [12], jump methods [13], and weighted gap statistics [14]. Moreover, supervised approaches to cluster validation such as using re-sampling strategy [15], prediction strength [16], methods based on mixture models and inference of Bayesian factors[17,18], or strategies which are application-specific [19] have also been previously implemented. Nevertheless, most existing methods are subject to certain hypothesis on a fixed dataset, and cannot be directly used for online phenotype discovery where new images continuously extend the dataset and millions of cells are involved. Improper assumptions on data structure may cause incorrect division or merging of biologically meaningful phenotypes. To avoid this problem, such methods combine each new image with the whole existing dataset (regardless of the large difference in cell numbers) and frequently re-run from the very beginning.

Methods for online phenotype discovery should be sensitive and flexible to various phenotypes and avoid frequent re-modelling involving complete existing datasets. As a kernel machine based novelty detection method, one-class SVM is used for "off-line" phenotype discovery [20]. However, two major points limit its application to high-throughput image-based screens, especially for screens of cell shape regulators. *First*, in one-class SVM all the test samples are classified into two classes, "novel" and "known", however many high-throughput RNAi datasets may potentially contain *multiple *diverse and unique novel phenotypes which should not necessarily be grouped together. Subsequent cluster analysis would be needed to identify and model different novel phenotypes following the use of one-class SVM. *Second*, each time a novel phenotype is discovered using one-class SVM, the support vectors need to be modified so that the newly discovered phenotype are included as "known" in the following loops, otherwise it will continuously be identified as novel in future. As mentioned earlier, in a typical RNAi screen on 1,000–10,000s genes with dozens of images for each RNAi and 100s of cells in each image, such updating would involve millions of cells and is intractable.

**Figure 1:**
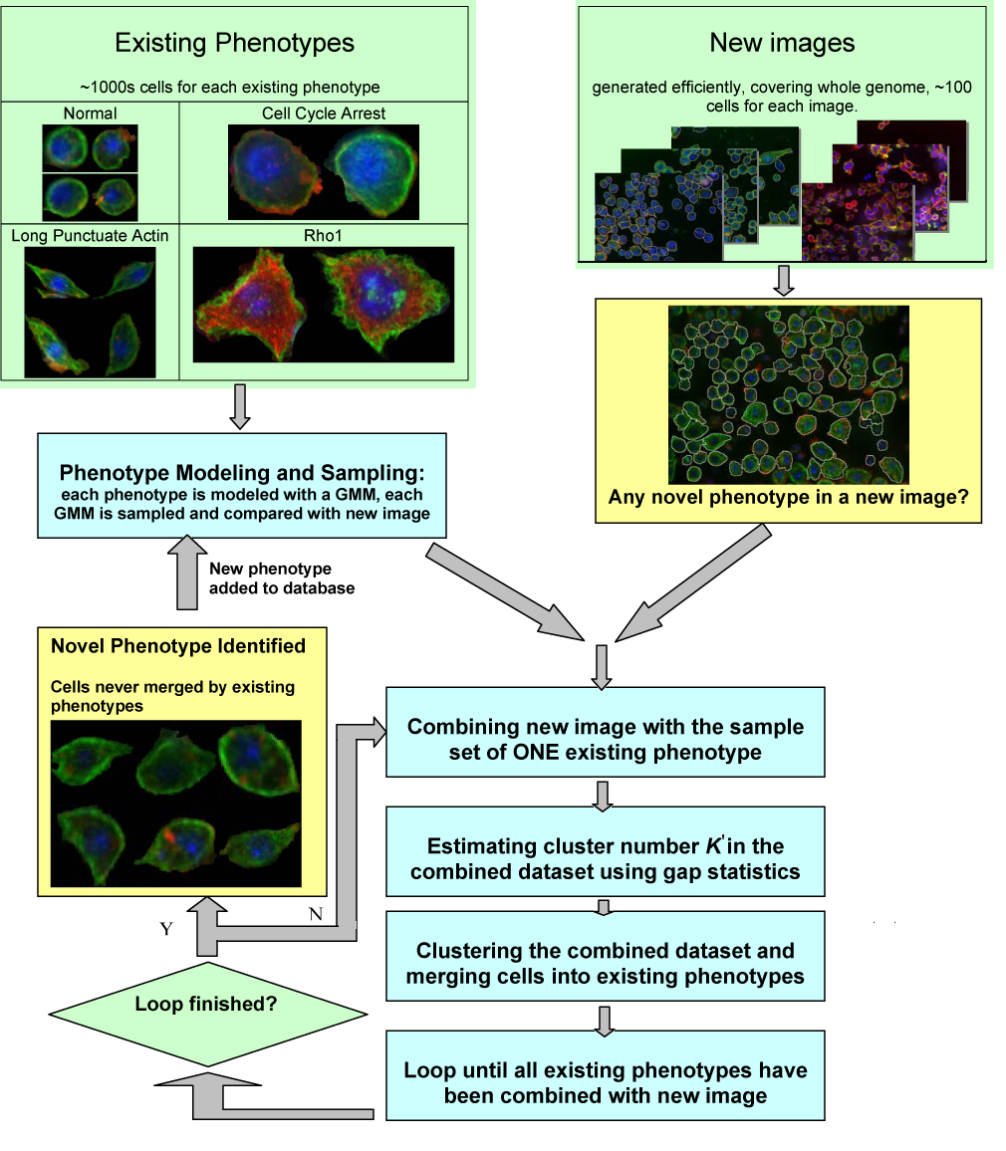
Tasks and simple scheme of online phenotype discovery.

Here we describe the development of an online phenotype discovery pipeline that we implemented in the context of a high-throughput image-based RNAi screen for regulators of cell shape. A simplified scheme of online phenotype discovery is shown in Figure 1. Online phenotype discovery demands adaptively identifying various novel phenotypes based on multiple existing phenotypes (e.g. those identified *a priori *by biologists), being sensitive and flexible to various new phenotypes and avoiding frequent re-modelling using large existing dataset. Our method includes two key components: phenotype modelling and iterative cluster merging. *First*, a Gaussian Mixture Model (GMM) is estimated for each existing phenotype following [21]. *Second*, iterative cluster merging are performed based on gap statistics. When a new image is incorporated, we sample the GMM of each existing phenotypes and start a series of merging loops. In each loop, the image is combined with sample set for one existing phenotype and we estimate cluster number in such combined dataset using gap statistics and use GMM of existing phenotype as part of the reference distributions. If some cells in the new image are clustered together with samples from the existing phenotype, they are merged into the existing phenotype, i.e. they are included into the dataset of existing phenotype and deleted from the new image. The iterations continue until sample set from each existing phenotype has been combined with the new image and has merged with its counterpart (if any exist). Upon completion of all loops, the remaining cell groups in the new image are identified as the candidate of new phenotypes. By sampling reference dataset from new image and existing phenotype separately, utilizing the GMM for existing phenotypes as (part of) reference distribution and involving existing clusters one by one, our method improves the ideas in [12] and becomes more effective. Experimental results show that the proposed method is robust and efficient for online phenotype modelling and discovery in the context of diverse image-based screens, especially RNAi screens on *Drosophila*.

## Results

### Synthetic dataset

#### Overcoming large sample size difference between two clusters

Difference between sample numbers of distinct clusters could bias cluster number estimation. We propose to tackle this problem by using GMMs as reference distributions for existing phenotypes in gap statistics and validate our method using simulation.

Each simulated dataset consists of observations from two populations *ℙ*_1 _and *ℙ*_2_, each population are sampled from a two dimensional Gaussian distribution with means (0,0) for *ℙ*_1 _and (0,3) for *ℙ*_2_, and an identity covariance for both groups. Gap statistics [12] are used to estimate number of clusters from the experiment dataset *ℙ*_1 _⋃ *ℙ*_2_. This method uniformly samples different reference datasets from the support of *ℙ*_1 _⋃ *ℙ*_2_, and here the number of reference datasets is set to 20. Then the experiment dataset *ℙ*_1 _⋃ *ℙ*_2 _and the 20 reference datasets are clustered into candidate cluster numbers *k *= 1... *K*, and we set *K *= 10. For each clustering result, we measure the compactness of obtained clusters using "within cluster dispersion". For each cluster number *k*, such dispersion are measured separately on experiment dataset and each reference dataset, and gap statistic for *k*, denoted as *gap(k)*, is defined as the average value of difference between the dispersion on experiment dataset and that on each reference dataset, meanwhile we obtained standard deviation of such difference across 20 reference dataset, and denoted as *s*_*k*_.

**Figure 2:**
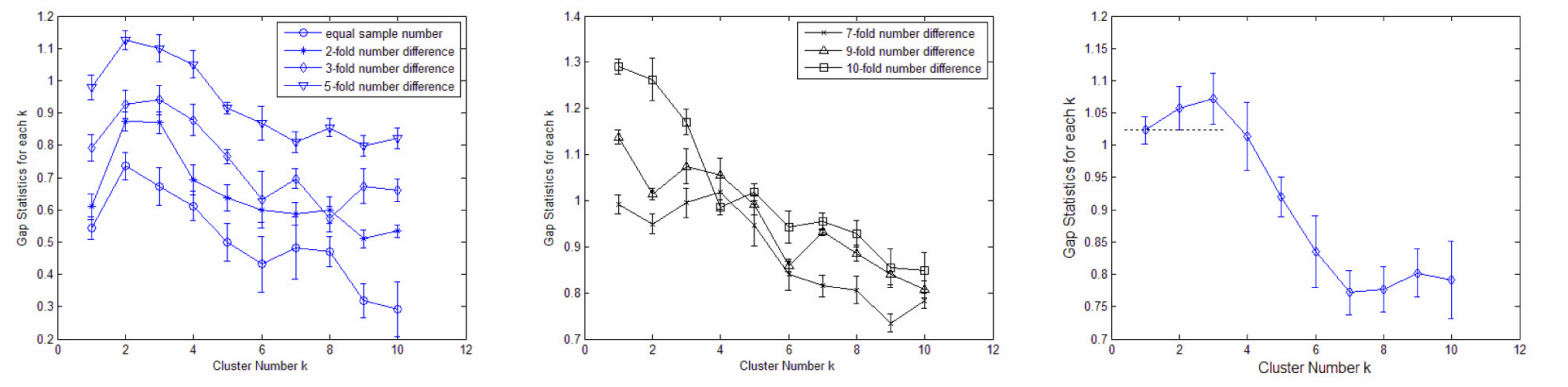
**Gap statistic curves for dataset with different sample number**. Each curve represents experiment on one real dataset, and twenty reference datasets are defined from this real dataset. For each data point, value on X-axis indicates how many clusters are defined on both the reference dataset and the real dataset and value on Y-axis indicates gap statistic for this cluster number, which is defined as the average difference of within cluster dispersions between the clustering results on reference datasets and real dataset, the error bars around the data points show the variation across different reference datasets. The estimated cluster number is defined as **the X value of the first data point with higher Y value than the bottom of error bar for its instant right neighbor**. During the experiments, the "real" dataset consists of two clusters and different reference datasets are used, *Left*, uniform reference distribution are used, sample number differences are equal, 2-fold, 3-fold and 5-fold from bottom to top, gap statistics works; *middle*, uniform reference are used, sample number differences are 7-fold, 9-fold and 10-fold from bottom to top, gap statistic fails; *right*, two clusters having 10-fold difference in sample number, Gaussian distribution is used as reference distribution for the cluster with larger sample numbers, and the cluster number is estimated accurately.

Some typical gap statistic curves are summarized in Figure 2 to illustrate the problem and validate our method. X axis in Figure 2 indicates candidate cluster number *i*, and each data point denote *gap(k) *while the error bar indicating *s*_*k*_. Larger value of *gap(k) *means better compactness when the dataset is clustered into *k *clusters (compared with the reference datasets which simulate mono-genous data), then a increase from *gap (k-1) *to *gap (k) *means the clustering performance is improved from *k-1 *to *k*. We take *s*_*k *_into consideration when estimating cluster numbers following [12], and take the estimated cluster number as the first *k *with *gap(k)>gap(k+1)-s*_*k*+1 _(the candidate number whose data point is higher than the bottom of error bar for its instant right neighbor). Details of gap statistics method are discussed in *Methods *section.

When samples from *ℙ*_1 _and *ℙ*_2 _have identical number of 100, gap statistics can correctly judge sample number as 2, then we set sample number from *ℙ*_1 _as 200, 300, 500, 700, 900 and 1000, and sample number from *ℙ*_2 _are fixed as 100. Figure 2, *left *and Figure 2, *middle *show that when number difference is over six-fold, gap statistics does not work correctly.

A ten-fold difference of cell numbers between existing phenotypes and new images is not the worst situation we would face in online phenotype discovery. Based on the results given in Figure 2, *middle*, samples from two obviously different populations would be merged together. We propose to solve this bias through fitting a GMM model for existing phenotypes, as well as using GMM as reference distribution for each existing cluster in gap statistics.

Next, we consider *ℙ*_1 _as existing cluster with known distribution model. If we use Gaussian model for *ℙ*_1 _as reference dataset for gap statistics, it gives the correct result in 87.4% occasions across 500 experiments even with ten-fold difference in sample number. Figure 2, *right *shows one gap curve with ten-fold difference, where *Gap(1)-(Gap(2)-s*_2_) = *-0.0006*, meanwhile *Gap(2)>Gap(3)-s*_3_, thus, the estimated cluster number is 2 rather than 3 (although *Gap(3)>Gap(2)*), because data point for *k *= 2 is higher than the bottom of error bar for *k *= 3.

The results are summarized in Table 1. Generally speaking, when two distinct clusters have ten-fold difference in sample numbers, original gap statistics method using uniform reference fails to estimate cluster number correctly, while our method of using accurate model as reference distribution can overcome this issue and give a correct estimation of cluster number.

**Table 1:** Cross validation results on overcoming the bias of cluster size difference. By using distribution models as reference distribution gap statistics can give correct result even under 10-fold difference.

Difference between sample number of *ℙ*_1 _and *ℙ*_2_	Average cluster number estimation accuracy % (Uniform reference distribution)	Average cluster number estimation accuracy % (GMM as reference distribution for *ℙ*_1_, uniform reference for *ℙ*_2_)
Equal	100	100
2-fold	88.5	98.1
3-fold	81.8	93.3
5-fold	69.2	91.0
7-fold	<20	89.5
9-fold	<20	88.9
10-fold	<15	**87.4**

#### Simulating typical cells using seven types of polygons

**Figure 3:**
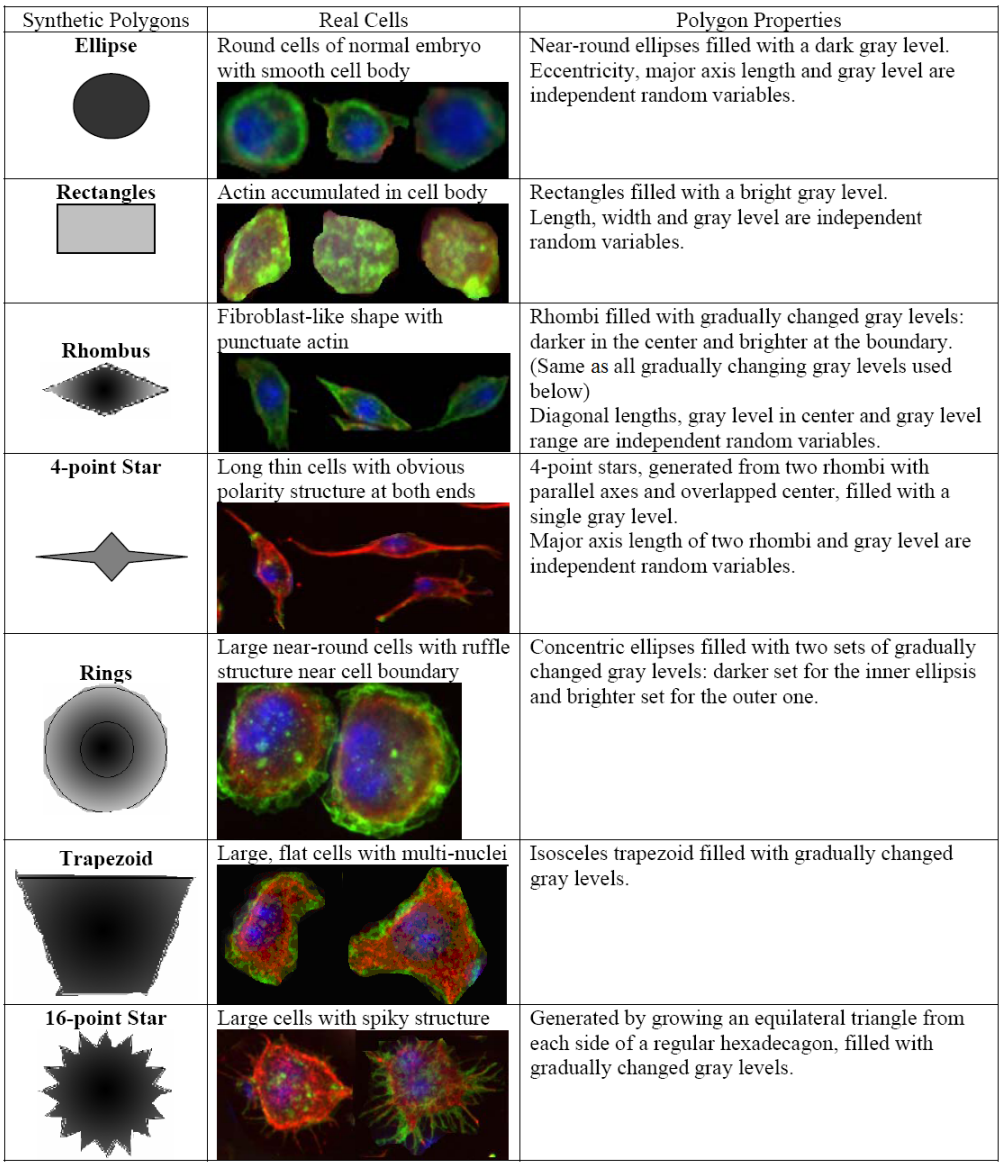
Information on seven polygon phenotypes used in simulation.

Seven types of polygons are defined based on the fluorescent cell image from a real genome wide RNAi screen (protocol discussed later), and a simulation dataset is constructed based on these different types of polygons. Information about these seven polygons is shown in Figure 3 and some details on generating these polygons are introduced in [Additional file 1].

2000 polygons from each of seven types were generated and used as training dataset, i.e. the set of existing phenotypes, and another 2000 polygons were generated for each type to as testing dataset. In each experiment, we started from a certain set of existing phenotypes and built GMM from training samples, meanwhile, we iteratively chose two of seven polygon types and selected 100 polygons apiece from the testing dataset to form a synthetic test image, altogether 70 images can be formed for one experiment. Using the model estimated from the training set, we can identify existing and novel type of polygons from these synthetic images, and observe the performance of our method under different number of novel phenotypes and order of image input.

#### Performance under different sets of existing phenotypes

**Figure 4:**
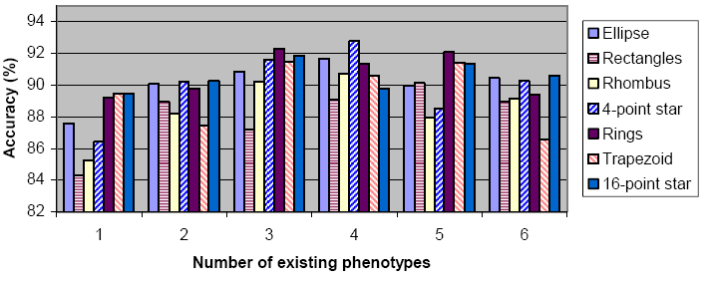
**Performance of our method on synthetic datasets with different sets of existing phenotypes**. For different number of "existing phenotypes" (X-axis), the performance on all seven types of polygons is summarized. Accuracy (Y-axis) indicates the ratio of test samples restored into its original clusters. All accuracy values are averaged across experiments with 50 different orders of image input and different composition of existing phenotypes (for number of existing phenotype 1–6, we have 7, 21, 35, 35, 21 and 7 different compositions, respectively).

Figure 4 shows the general performance of our method under different sets of existing phenotypes. We changed the number and composition of existing phenotypes for different experiments, (different composition means different phenotypes are considered as "existing". GMM for existing phenotypes were estimated from training dataset to start each experiment), and for each set of existing phenotypes, we divided the testing set into 70 synthetic images and shuffled the order of image input 50 times. Whatever set of existing phenotypes we use, the synthetic images always contain all seven phenotypes. We can define accuracy for each polygon type as "the proportion of test samples restored into their original cluster". If one phenotype is used as existing phenotype in an experiment, the accuracy for this phenotype is defined as the proportion of testing cells (in this phenotype) merged into the original existing cluster; while if the phenotype is novel, the accuracy for this phenotype equals the proportion of testing cells in this phenotype which are left alone in a separate cluster after all the merging loops. The accuracies are then averaged to report general performance of our method.

Figure 4 shows our method as having consistent accuracy around 85% for different polygons under different conditions, and the best performance is seen when the number of existing phenotypes are 3 and 4. When the number of existing phenotype is 6, more false negatives appears as novel samples are merged into existing phenotypes, and thus prompts the importance of cluster validation and more refined multiple hypothesis tests.

#### Box and whisker plots for performance under different conditions

**Figure 5:**
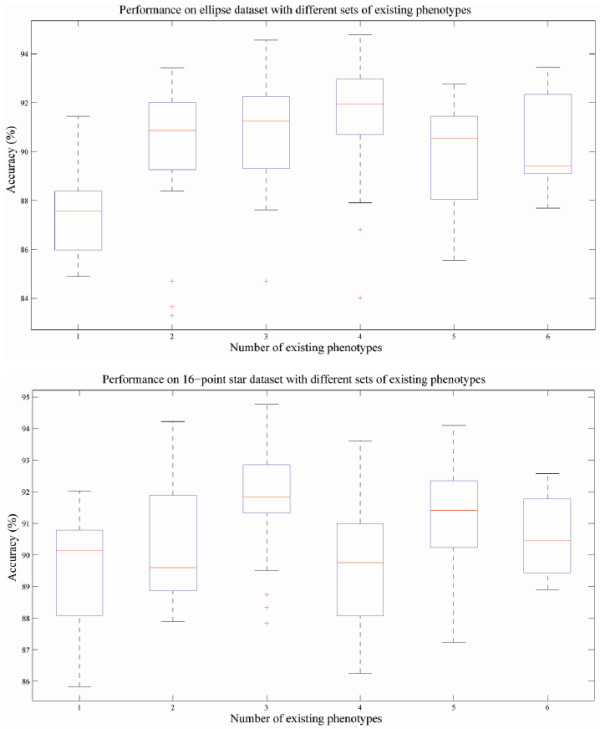
**Box and whisker plots indicating the robustness of performance under different condition**. The accuracy of each experiment is sorted in descending order and plotted on the Y-axis, the two horizontal edges of boxes indicate upper and lower quartile of accuracy values while the red line in the box body shows the median value. The whiskers and lines extending from the end of boxes show the extent of the rest data, and red crosses (+) are outliers with accuracy values beyond 1.5 times of inter quartile range. The performances on two polygon types are shown. Accuracy values of different experiments with different image input order but the same number of existing phenotypes are summarized in box and whisker plots. *Upper*, performance on ellipses phenotype; *Lower*, performance on 16-point stars phenotype.

As indicated earlier, the test datasets always consist of seven phenotypes, we can then change the conditions of experiments (number and composition of existing phenotypes and order of image input) and observe the performance for certain phenotype to test the robustness of our method. Across experiments with different input order of images and composition of existing phenotypes, the performance on certain phenotypes are ranked, and such distribution of performance are illustrated using box and whisker plot. We show the box plots for ellipses and 16-point stars in Figure 5 and explain the meaning of such plots in its caption. We observed that larger variation of accuracy values correlates with lower number of existing phenotypes, which is because the models of novel phenotypes are estimated from test samples input in the earlier stage of experiment and updated as new images are included. Thus, the accuracy has a larger variance across different image input order when the number of existing phenotypes is low. This issue indicates the importance of more robust strategy of model updating. However, the overall performance of our method is robust regarding to different condition of experiments.

#### Performance comparison with SVM based methods

One-class SVM [20] tackles the novelty detection problem of differentiating novel phenotypes from known ones by estimating a distribution from the core structure of existing dataset and model such distribution using a series of support vectors. It then labels each testing samples as "known" or "novel" using the model built upon support vectors. Compared with SVM used in classification, a parameter *ν *∈ (0, 1) (denoted as 'Nu' in figures) is involved in one-class SVM. This parameter is used to define the core structure of the existing dataset, and it has two roles, i.e. the asymptotic upper bound of training data which are labelled as outliers and the lower bound of the fraction support vectors in training samples. However, one-class SVM itself cannot be used to handle problems such as the restoration of multiple existing phenotypes. We modify one-class SVM to fit it into the scenario of online phenotype discovery. Each new image is combined with the support vectors trained from the existing samples and novelty detection is carried out using one class SVM with Gaussian kernels of width 0.5 and various parameters *ν *to define the scale of support vectors and outliers. After novelty detection, each test sample labelled as "known" is subject to multiple linear SVM classifiers (trained from one pair of existing phenotypes) and assigned into one of multiple existing phenotypes according to majority vote among classifiers. We detailed one-class SVM and our modification in [Additional file 1].

**Figure 6:**
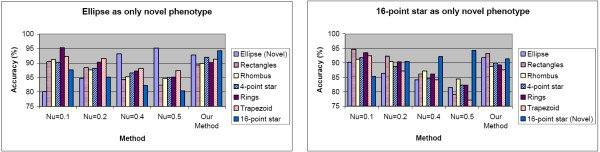
**Performance comparison between our methods and SVM based methods on two occasions**. In each experiment, six polygon types are used as existing phenotypes. Accuracy denotes the ratio of samples restored to its original phenotypes. All the accuracy values are averaged across 100 tests having different order of image input. Four different sets of parameters are used for SVM based method. *Left *Ellipses serve as novel phenotype, the other six serve as existing phenotype; *Right *16-point stars serve as novel phenotype.

We compared our method and SVM based method and Figure 6 shows the comparison results in two occasions. Four sets of parameters are used for one-class SVM. Figure 6*left *shows the performance when ellipses are considered as novel phenotype while the other six phenotypes serve as the set of existing dataset; and Figure 6*right *shows the performance when 16-point stars are the only novel phenotype. Accuracies for all seven phenotypes were measured and averaged across 100 experiments with different orders of image input. In both cases, when *ν *= 0.1, SVM based method merged most of the new samples into existing phenotypes and gave best performance on existing phenotypes (especially for 16-point stars), while SVM with *ν *= 0.5 left out most of the new samples as outliers and gave the best accuracy for the novel phenotype, meanwhile, the difference between best and worst accuracy in one experiment could be as large as 25%; our method outperformed most SVM based method on the accuracies for at least 3 of 7 phenotypes and the difference between best and worst accuracy across seven types are never greater than 10%.

### Cell culture, image segmentation, morphological feature extraction and selection

#### Cell culture and image acquisition

As a next step, we implemented our methods in the context of a high-throughput image-based screen. In particular, we focused on a novel dataset of images acquired in the course of a genome-scale RNAi screen for regulators of *Drosophila *Kc167 cell shape that have hemocyte-like properties (Bakal et al, unpublished). By using dsRNA to target and inhibit the activity of specific genes/proteins, the role of individual genes in regulating morphology can be systematically determined. Briefly, Kc167 cells are bathed in the presence of individual dsRNAs targeting all known *Drosophila *protein kinases and phosphatases in 384-well plates (detailed protocols are available at [22]). Following a 5-day incubation period, the cells are fixed and stained with reagents in order to visualize the nuclear DNA (blue channel in all images), polymerized F-actin (green), and *α*-tubulin (red). For each well, sixteen images from each of the three channels (blue, green and red) were acquired in an automated fashion using an Evotec spinning-disk confocal with a 60× water objective. Auto-focusing is performed in a two-step fashion by first focusing on the bottom well at each individual site, and then moving the objective by the same Z-distance (in this case 3 *μ*m above the bottom of the well) at each site. The images were captured at a binning of 2 and have a resolution of 661*481 pixels.

#### Image segmentation

To analyze the morphology of single cells, it is necessary to first delineate the boundaries of individual cells. Direct segmentation of the cell bodies in the F-actin and *α*-tubulin channels is difficult due to the complex morphology of cellular boundaries. Segmentation of nuclei in the DNA channel is relatively easier, and its segmentation results provide the rough position information of the cell bodies. Herein, we utilize a two-step segmentation procedure [5,23] including nuclei segmentation on DNA channel, and cell body segmentation of images derived by combining images from the DNA, F-actin and *α*-tubulin channels.

In nuclei segmentation, the nuclei are first separated from the background by using a background correction based adaptive thresholding method [24]. However, the clustered nuclei cannot be separated by the adaptive thresholding method. To separate the clustered nuclei, the centres of the nuclei are first detected using a gradient vector field (GVF) based detection method [24]. Specifically, we filter the nuclei image using a Gaussian filter, which suppresses the noise and generate local maxima inside cells, and these local maxima correspond to the nuclei centres. However, there are still some local maxima due to noise. To further eliminate the noisy local maxima, we detect the true cell centres using GVF method. It is a well-known fact that in an electric field, the electric field lines point to the positive electrodes, and the free negative electrons move along the electric field lines and stop at these electrodes. In GVF, the gradient-vector lines also point to the local maxima. Analogous to the electron moving inside the electron field, we put one particle on each detected cell pixel and pushed it along the gradient vector lines. Consequently, these particles stop at these local maxima. Since no or very few particles stop at non-maxima and noisy local maxima, the true cell centres can be identified by choosing the points that have many particles [24]. After the centres of nuclei are detected, the nuclei are segmented using the marker-controlled watershed algorithm.

To use both F-actin and *α*-tubulin channels information, we combine the two channels' signal as I = (I_F-actin _+ I_*α*-tubulin_)/2, where I, I_F-actin _and I_*α*-tubulin _denote the combined image, F-actin channel image and I_*α*-tubulin _channel image respectively. We then segment the cell bodies using the combined image. First the cell bodies are separated from the background using the aforementioned adaptive thresholding algorithm. The nuclei segmentation results facilitate the segmentation of cell bodies by providing the rough position information of cell bodies. Herein, we employ the marker-controlled watershed and the nuclei segmentation results to segment the individual cell bodies. To reduce the over-segmentation of cell bodies a feedback system proposed in [5] is employed. Three scoring models, which measure the morphological appearance, gradient and edge intensity of cell pairs respectively, are built to identify the over-segmented cell bodies, and guide the merging procedure [5]. Detailed shape and boundary information of nuclei and cell bodies is obtained after the two-step segmentation procedure.

#### Morphological feature extraction and feature selection

Cellular phenotype identification depends on choosing a rich set of descriptive features, which is one of the most critical steps for pattern recognition problems. To capture the geometric and appearance properties, 211 morphology features belonging to five categories are extracted following [23]. The selected features include a total of 85 wavelet features (70 of them from Garbor wavelet transformation [25] and 15 features from 3-level CDF97 wavelet transformation [26]), 10 geometric region features describing the shape and texture characteristics of cells [23], 48 Zernike moments features with selected order of 12 [27], 14 Haralick texture features [28] and a total of 54 phenotype shape descriptor features (36 features of ratio length of the central axis projection and 18 features of area distribution over equal sectors) [23]. A feature selection procedure is necessary to de-noise the dataset and describe it in the most informative way. As the datasets and phenotype models are being updated adaptively, an unsupervised feature selection without relying on phenotype labels is used to supply a stable feature subset. It is based on iterative feature elimination using *k *nearest neighbour features following [29]. In this study, an informative subset of fifteen features is selected to quantify the segmented cells.

### Online phenotypes discovery in the context of RNAi high-throughput screenings

#### Fitting GMM model for existing phenotypes

**Figure 7:**
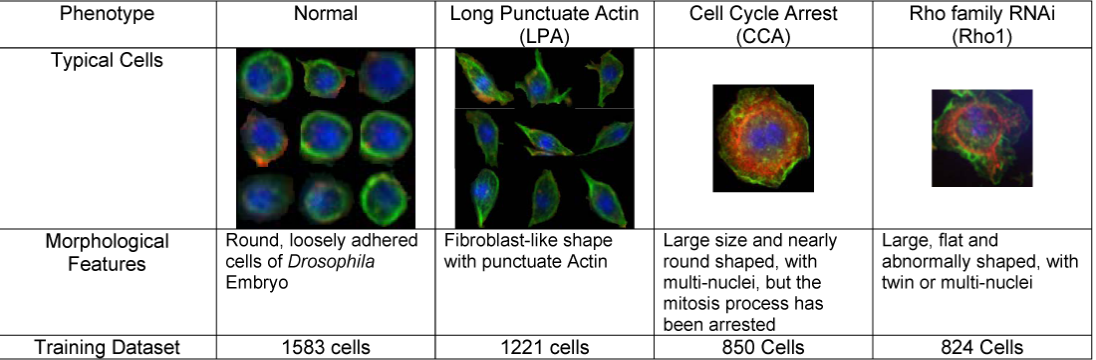
Information of four existing phenotypes in training dataset.

We first performed a visual examination on a subset of dataset in order to define images and cellular morphologies of typical normal cells, as well as cells in three distinct cellular phenotypes. We termed these quantitative categories "Long Punctuate Actin (LPA)", "Cell Cycle Arrest (CCA)" and "Rho1 (Rho)", collected images in these categories and combined them with images of 1583 normal cells, to form our cell database of existing phenotypes. We estimated a GMM for each existing phenotype using EM algorithms. A uni-modality model is obtained for normal and LPA phenotypes, and CCA and Rho phenotypes end up with 2 and 3 Gaussian terms respectively and the covariance matrices are set to be diagonal. A brief introduction of each existing phenotype is shown in Figure 7.

#### Case 1: merging cells in existing phenotypes

To validate our method's ability of restoring three existing phenotypes: Normal, LPA and CCA, we carried out 100 times of five-fold cross validation. In each experiment, 20% of cells from each existing phenotypes were taken out and combined to form a flow of new images, these cells were divided into seven groups, simulating seven new images. We defined merging accuracy for each phenotype as the proportion of test samples merged into its original phenotype. The mean and standard deviation of accuracies across 100 experiments was shown in Table 2.

**Table 2:** Cross validation results on merging cells into existing phenotypes

Phenotype	Average merging accuracy and standard deviation %	Typical Mistakes
Normal	98.6 (1.7)	left alone
LPA	93.8 (2.9)	Merged into Normal, left alone
CCA	92.4 (2.4)	left alone

Our method can identify and merge cells into original phenotypes well. In the third column of Table 2, we list the typical mistakes made during the merging loops. Some cells with normal and LPA phenotypes are not merged correctly, and such mistakes suggest the existence of previously undefined phenotypes in such images.

#### Case 2: discovering new phenotypes: cross validation based on known phenotypes

To validate our method's ability of discovering new phenotypes, we performed the following experiments after the *a priori *identification of four existing phenotypes (Normal, LPA, CCA and Rho1) by biologists. In each experiment, three of four phenotypes were considered as existing phenotypes, and cells in the other phenotype were divided into groups with 100 cells. These cell groups represent an incoming dataset of new images containing "novel phenotypes". Given only one phenotype in the testing images, ideally in each experiment, our method would identify the first of such cell groups as a new phenotype, model this phenotype, assign all the other testing images to this "novel phenotype", and no testing cells would be merged by any of the other three phenotypes. Fifty experiments were performed to identify each phenotype, 1000 cells served as "novel group" for normal and LPA phenotypes while 600 cells were used for CCA and Rho1 phenotypes. The experiment results are summarized in Table 3. "Accuracy" is defined as the ratio between the number of testing cells assigned to a single cluster and the total number of testing cells, and such accuracy is calculated after all three merging loops for each experiment.

**Table 3:** Cross validation results on discovering new phenotypes

Phenotype to be identified	# of cells to be identified	Average accuracy with standard deviation %	Typical Mistakes
Normal	1000	95.2 (3.5)	Left alone
LPA	1000	90.3 (3.1)	Merged into Normal, left alone
CCA	600	89.6 (2.7)	Merged into Rho1
Rho1	600	87.4 (4.2)	Left alone

Case 2 shows our method's ability of identifying novel phenotypes. We hypothesize that the relatively low accuracy for CCA and Rho phenotypes can be attributed to the small number of samples and incomplete understanding of which phenotypes is the biological representative for the entire treatment class. High classification accuracies for normal cells in both case 1 and case 2 provide strong validation of the ability of our methods to identify wild-type cells. While the overlap of normal and LPA serves as a starting point for novel phenotypes discovery.

#### Case 3: identifying multiple novel phenotypes from online image input and performance comparison with SVM based methods

In this case, we still used the test dataset in case 2, which included a total of 3,200 cells from four phenotypes. In each group of experiments we started from the models (available from previous step) of two existing phenotypes, and all 3,200 test cells were divided into 32 images with cells from two phenotypes in one image, and all images were input with 50 different orders. Altogether, three groups of experiments were carried out, and in each experiment, normal phenotype were paired with one of the other three phenotypes, to serve as sets of ''existing phenotypes''. Both our method and SVM based methods were used in each experiment, and we can thus validate our method's ability to deal with multiple novel phenotypes well as the performance under different order of image input.

**Figure 8:**
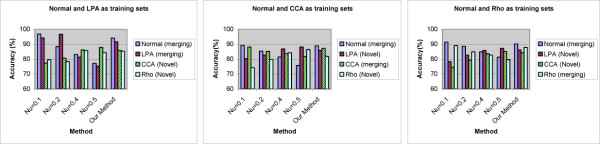
**Performance comparison between our method and SVM based methods with multiple phenotypes in images**. Given certain group of existing phenotypes, and images including multiple phenotypes, the accuracy values for four phenotypes across 50 image input orders are shown. Four different sets of parameters are used for SVM based method. *Left *Normal and LPA as existing phenotypes; *Middle *Normal and CCA as existing phenotypes; *Right *Normal and Rho as existing phenotypes.

Figure 8 summarizes the average performance of our method and SVM based methods for each phenotype across 50 experiments. The results from different sets of existing phenotypes are shown separately, and "accuracy" is defined as the proportion of test samples restored into its original cluster. The performance of our method degraded with reduced number of existing phenotypes compared with case 1 and case 2, especially for the Rho1 phenotype, however, it still performed consistently on existing and novel phenotypes, never failed to reach 80% mark and outperformed SVM based method in at least two of four phenotypes on all occasions.

**Figure 9:**
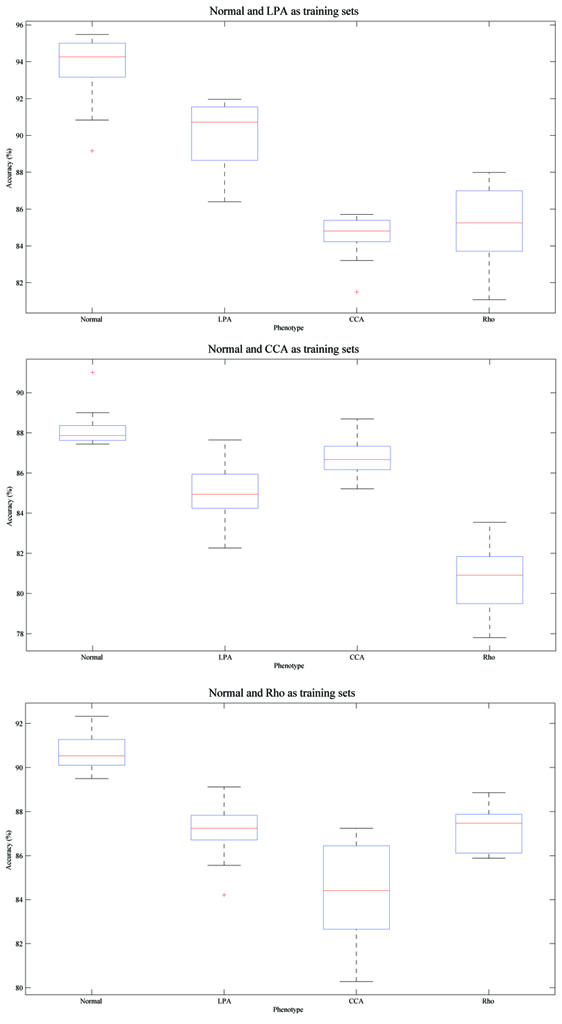
**Box and whisker plots indicating the robustness of performance with multiple phenotypes in images**. The accuracy of each experiment is sorted in descending order and plotted on the Y-axis, the two horizontal edges of boxes indicate upper and lower quartile of accuracy values while the red line in the box body shows the median value. The whiskers and lines extending from the end of boxes show the extent of the rest data, and red crosses (+) are outliers with accuracy values beyond 1.5 times of inter quartile range. Given certain group of existing phenotypes, and images including multiple phenotypes, the accuracy values for four phenotypes across 50 image input orders are shown. *Top *Normal and LPA are existing phenotypes; *Middle *Normal and CCA are existing phenotypes; *Bottom *Normal and Rho are existing phenotypes.

Figure 9 shows the box and whisker plots for our methods under three different sets of existing phenotypes. The variation of accuracy across different order of image input can be as large as 8% (Figure 9*bottom*, accuracy for CCA phenotype), but the accuracy is never less than 80% For two experiment with the lowest accuracies and shown as outlier in the box and whisker plot (Figure 9*top*, CCA and Figure 9*bottom*, LPA), the order of image input was validated, and on both occasions we observed a small group of novel cells emerging at the beginning of image flow with all their counterparts appearing very late. Thus, the model for novel phenotypes was only built based on a small group of cells, and most test cells appeared later were merged by similar phenotypes with more stable models. This indicates the necessity of better cluster validation and model updating solution, which are proposed in our method.

Having multiple phenotypes in a single image is a challenge in the analysis of image based high-throughput screens. Our method successfully tackle such cases and identify multiple phenotypes from online image input, and can therefore provide a better perspective for further quantification of the whole image, *en route *to the identification for the role of each gene.

#### Identifying "rl/*tear-drop*" phenotype in the context of Drosophila genome-scale RNAi screen

**Figure 10:**
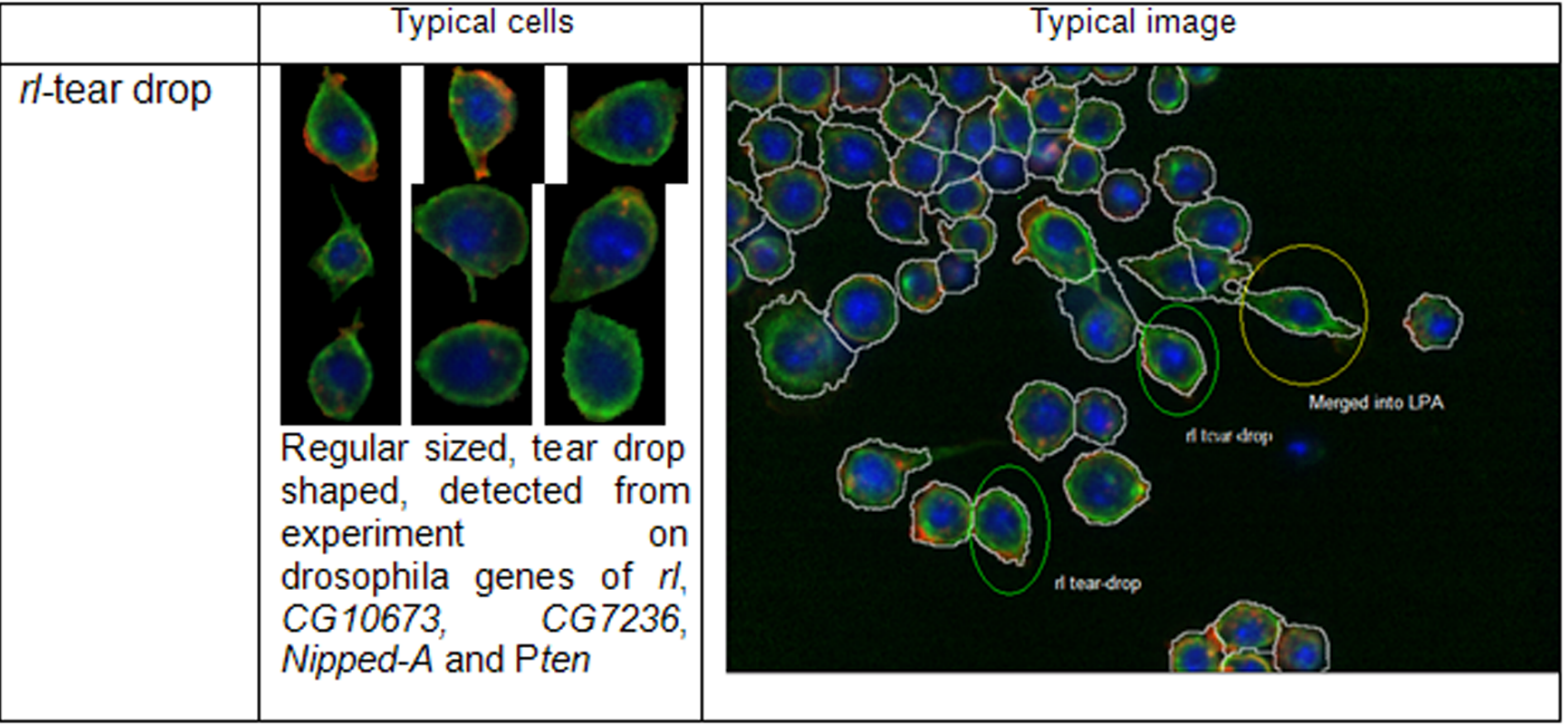
**Information for the *rl*/tear-drop phenotype**. "Typical cells" summarizes the properties of the *rl*-tear drop phenotype and "Typical image" shows an image with cells merged by *rl*-tear drop, LPA and Normal phenotype respectively.

Typically, a new image which is incorporated into the dataset may have less than fifty cells, which can severely impact the ability to quantitatively determine and distinguish novel phenotypes. But such images can still be incorporated into our analysis by discarding images with cell numbers <10, and putting together cells from multiple new images to make a combined dataset having at least 100 cells. We collected cells from four existing phenotypes, assembled them as training dataset, modelled existing phenotypes and used such phenotype models to analyze images acquired as part of a *Drosophila *genome-scale RNAi screen. Implementation of our method revealed a phenotype that was undetected by visual inspection which we termed as "*rl*/tear-drop". These cells are small in size and having smooth boundaries and non-round shape. Figure 10 supplies some information and typical images for this phenotype. Such phenotype was initially discovered in wells where cells had been incubated in the presence of *rl *RNAi. *Drosophila rl*, or *rolled *is the homolog of mammalian ERK kinase and is a central regulator of a host of cellular processes [30]. Importantly, the *rl*/tear-drop phenotype was not scored during human inspection of the images. The detection of cells with the *rl*/tear-drop phenotypes in an image or well often correlates with detection of cells with an LPA phenotype, and we hypothesize that *rl*/tear-drop represents a phenotypes that occur as cells transition from normal to LPA. We collected more than 500 cells with the *rl*/tear-drop phenotype through analysis of replicate experiments, modelled this phenotype with GMM, and used it to analyze cells from the new images. Our method detects *rl*/tear-drop cells in experiments where *CG10673, CG7236, Nipped-A *and *Pten *have been targeted by RNAi. Although the nature of the relationship among these genes needs further investigation, we have previously identified *Nipped-A *and *Pten *as regulators of ERK activity following insulin stimulation [2], suggesting a relevant relationship between these genes. Altogether, these results demonstrate that our online phenotype discovery methods can be used to provide unexpected and novel biological insight.

We also tested our method using two published dataset from *Drosophila *RNAi screen [7] and HeLa cell cycle phase detection [*ref*. s3 in Additional file 1], compared its performance with SVM based methods and validated our method's ability of handling dataset from various organisms. These experiments are described in [Additional file 1], the results on *Drosophila *dataset from [7] are reported in [Additional file 2] and results on HeLa dataset are reported in [Additional file 3, 4].

All other functions are developed in Matlab 7.0 and ran in PC with Intel^® ^Core™ 2 T7200 2.00 GHz CPU and 2.00 GB of RAM. Starting from four existing phenotypes, the average running time for our method based on improved gap statistics is 1.8 seconds on a group of 100 segmented cells, 10.2 seconds on a group of 600 cells and 19.4 seconds on a group of 1000 cells. Considering the fact that cell number in each image is seldom over 300 in the reported high content screen, our method is suitable for online application.

## Discussion

Online identification and validation of novel morphological phenotypes are major challenges in specific high-throughput image-based screens. Manual phenotype labelling of high-throughput image-based data is a laborious and inordinately time-consuming process, while available automatic identification methods usually classify cells into a limited set of predefined phenotypes which may be determined through biased means and will not be updated according to the online image input. As millions of images are now generated during the course of a comprehensive genome-scale screen, new methods are needed to effectively identify novel phenotypes in such massive databases. Here we report the development of an online phenotype discovery method which models existing phenotypes, compares cells in new images with existing phenotype models through cluster analysis, assigns some new cells to existing phenotypes, and finally identifies and validates novel phenotypes online.

GMM is used for modelling existing phenotypes and gap statistics, with GMM as reference distribution for existing phenotype, plays a key role in cluster analysis and merging. We built GMM for existing phenotypes, sampled datasets from the model and used them as reference distribution in gap statistics method, following this pipeline we can cover the complete properties of phenotypes more efficiently. Furthermore, gap statistics are dealing with only one existing phenotype plus a part of the new image in each merging loop, and the content of new image is iteratively updated with the merging procedure. We present Additional file 5 to validate the idea of modeling existing phenotype using GMM, the detailed information of GMM estimated from four existing phenotype in our real dataset are reported along with histograms for some typical feature. 

For analysis of high content screen data, many researchers choose to summarize the information of single cells or objects to supply a normalized signature for higher level concepts (e.g. treatment conditions, genes, complexes, etc). Thus it is critical to identify different phenotypes related to a same treatment condition. Our method can be used in identifying multiple phenotypes in single well and supply detailed insight into related questions. The performance of our methods relies on the quality of image processing, feature selection, phenotype modelling, and cluster analysis methods. Using iterative cluster merging, our future goals are to build more reliable phenotype models and to construct complete pipelines of cluster analysis with detailed validation procedures to obtain more reliable definition of clusters.

## Conclusion

We propose an online phenotype discovery method for high-throughput RNAi screen, which can be used in the course of many image-based screens. This method is based on adaptive phenotype modelling and iterative cluster merging using improved gap statistics. Given datasets for existing phenotypes, the method can build a model of each existing phenotype, identify novel phenotypes in images obtained from ongoing screening and assign newly obtained cell images into different phenotypes. Compared with traditional novelty detection techniques, our approach avoids frequent re-modelling involving the huge existing dataset and can handle multiple existing phenotypes in a flexible manner. Implementation of our methods in the analysis of images acquired during a genetic screen for regulators of *Drosophila *cell morphology demonstrates the power of these computational tools in efficiently discovering meaningful new phenotypes.

## Methods

### Online cluster discovery: problem formulation

Suppose we have identified *K*_0 _non-overlapping cellular phenotypes, the *i*-th cell in the *m*-th existing phenotype is denoted by vector $s_{i}^{\left(m\right)}≜[s_{i,1}^{\left(m\right)},s_{i,2}^{\left(m\right)}...s_{i,p}^{\left(m\right)}]$, with each cell described by *p *morphological features. Then, let $S_{m}≜{\left\{s_{i}^{\left(m\right)}\right\}}_{i=1}^{u_{m}}$ denotes dataset for the *m*-th phenotype, with *u*_*m *_indicating the number of cells for *m*-th phenotype. Thus, we denote the dataset of all available cells, S, as:

(1) $$\begin{array}{l} S=\overset{K_{0}}{\underset{m=1}{\cup }}S_{m}\\ \text{s}\text{.t}\text{. }\forall m,n\in \left\{1,2...K_{0}\right\},S_{m}\cap S_{n}=\emptyset \end{array}$$

and the total number of existing cells is $u=\sum_{m=1}^{K_{0}}u_{m}$. Similarly, when a new image E is obtained, the *i*-th cell in this image is also described using *p *features, and denoted by **e**_*i *_≜ [*e*_*i*1_, *e*_*i*2 _... *e*_*ip*_], and $E={\left\{e_{i}\right\}}_{i=1}^{v}$, where *ν *is the number of cells in E. New images are continuously obtained, and each new image E contains tens of cells while there are thousands cells for each S_*m*_, thus *v *<<*u*_*m *_<*u*.

Given a new image E, we need to adaptively determine number of new phenotypes *K*_*new*_, based on *K*_0_, S and E. Cells in E while belonging to some existing phenotype S_*m *_should be identified, and used to update model for S_*m*_. It is unfeasible to involve every single cell in S into cluster discovery, because the large scale of S could bias cluster analysis towards existing phenotypes and add computation burden. On the other hand, "new cluster" identified only according to E is vulnerable to outliers. Thus an efficient method to utilize S is necessary.

### Outline of the proposed approach

We propose to discover new clusters through iterative cluster merging. The dataset of each existing phenotype S_*m *_is first fit to a GMM and sample dataset $S_{m}^{'}$ is obtained from such model. Each $S_{m}^{'}$ is combined with a new image E one by one to detect possible new phenotypes. Our online phenotype discovery method is outlined as below:

(1) Phenotype modelling. A GMM is fit to each existing phenotypes using Expectation-Maximization (EM) algorithm following [21].

(2) Sampling existing phenotype and combining existing information with the new image. We sample from the GMM of one existing clusters, say S_*m*_, *m *∈ {1, 2... *K*_0_}, get the sample set $S_{m}^{'}$, and put $S_{m}^{'}$ together with new image E, we denote this combined set as F, thus

(2) $$F=S_{m}^{'}\cup E$$

$S_{m}^{'}$ should have comparable cell numbers as *ν*, the cell number in E, so that phenotype information would not be overwhelmed due to limited cell number of E. We empirically set sample number of $S_{m}^{'}$ as *ν *to 5*ν*.

(3) Estimating the cluster number in F. An improved gap statistics method is used in which we take reference dataset from the range of feature values of $S_{m}^{'}$ and E separately, and use GMM as the reference distribution for reference samples obtained from the support of $S_{m}^{'}$.

(4) Defining clusters on F. Based on the estimated cluster number from step 3, a partition of F is obtained using Partitioning Around Medoids (PAM) [31] method.

(5) Merging samples from E to existing phenotypes.

(a) If some samples from E are assigned to a same cluster as at least 95% samples from $S_{m}^{'}$, they are considered as a candidate for merging.

(b) Validate merging operation using a statistical test with Bonferroni correction. For each merging candidate, calculate its *p *value under the GMM for S_*m*_, reject the merging operation and keep this sample in E if *p *value is smaller than 1/*K*_0_, or else merge this candidate into S_*m *_and delete it from E.

(6) Returning to step 2 to sample another existing phenotype and start new merging loop.

(7) Updating phenotype models. After each existing phenotype S_*m*_, *m *∈ {1, 2... *K*_0_}merges their counterparts in E, define clusters left in E as new phenotypes and estimate GMM for them.

Through modelling and re-sampling, S becomes more flexible and re-useable, allowing us to cover complete properties of phenotypes. Following data modelling and sampling, the information from existing phenotypes is combined with new image one by one in the loops from step 3 to 5. Thus in each single loop, the task of estimating cluster number is simplified to identifying difference between new image E and only one existing phenotype. After each S_*m *_merges its counterpart in E, clusters left in E are identified as new phenotypes.

### Cluster modelling and sampling

Given the dataset S, we model each phenotype S_*m *_using a GMM:

(3) $$\begin{array}{l} S_{m}∼\sum_{t=1}^{Q_{m}}\pi_{m,t}N(\pi_{m,t},\Sigma_{m,t}),m=1,2,...,K_{0}\\ s.t.\sum_{t=1}^{Q_{m}}\pi_{m,t}=1,\pi_{m,t}\geq 0 \end{array}$$

where *N *denotes Gaussian distribution. We denote the number of Gaussian terms for phenotype S_*m *_as *Q*_*m *_and define parameters for S_*m *_as $\pi_{m}={\left\{\pi_{m,t}\right\}}_{t=1}^{Q_{m}},\mu_{m}={\left\{\mu_{m,t}\right\}}_{t=1}^{Q_{m}},\Sigma_{m}={\left\{\Sigma_{m,t}\right\}}_{t=1}^{Q_{m}}$. Initially, the covariance matrix **Σ**_*m*, *t *_is set to be diagonal. We use Expectation-maximization (EM) algorithm to estimate {**π**_*m*_, **μ**_*m*_, **Σ**_*m*_} from S_*m*_. In the initialization of EM algorithm, *Q*_*m *_is set to four, and S_*m *_is first partitioned into *Q*_*m *_clusters using fuzzy C-means clustering method, and then initial parameters are estimated using the standard vector quantization method. For each class, *Q*_*m *_is reduced to the minimum possible using minimum description length (MDL) technique, following [21]. We obtain random samples from the GMM to form set $S_{m}^{'}$ having *i.i.d *S_*m*_, $S_{m}^{'}$, and $S_{m}^{'}$ is combined with new image E to form F. When estimating cluster number in F using gap statistics, GMM is used as reference distribution for $S_{m}^{'}$.

### Estimating cluster numbers using improved gap statistics

To estimate the number of clusters from an unlabeled dataset, many existing methods focus on the within cluster dispersion *W*_*k*_, resulting from clustering datasets (e.g. F) into *k *clusters, *C*_1_, *C*_2_,... *C*_*k *_with *C*_*r *_denoting the indices of samples in clusters *r *and *f*_*i*, *j *_denotes the value of *j*-th feature measured from *i*-th data point. Based on $D_{r}=\sum_{i,i^{'}\in C_{r}}\sum_{j=1}^{p}{\left(f_{i,j}-f_{i^{'},j}\right)}^{2}$, we have $W_{k}=\sum_{r=1}^{k}\frac{1}{2n_{r}}D_{r}$. *W*_*k *_tends to decrease monotonically as the number of clusters *k *increases, but from some *k *on, such decrease flattens markedly. Statistical folklore has it that error measure based on *W*_*k *_should have an "elbow" at the desirable cluster number, thus different criterions based on *W*_*k *_are defined.

Gap statistics [12] method utilizes the output of any clustering algorithm under different *k*, compares the change of *W*_*k *_to the dispersion expected under a reference null distribution, the gap between the logarithms of these two dispersions are employed to detect cluster number. Gap statistics can detect homogenous non-clustered data against the alternative of clustered data [14]. This ability is critical when all cells in new image E belong to the same phenotype.

To estimate cluster number in F (defined in equation (2)), we first select a number *K *which is larger than the expected cluster number, e.g. in our case *K *= 5. For each *k *= 1, 2... *K*, F is divided into *k *clusters and gives a series of *W*_*k*_. Then we generate *B *reference datasets from F, cluster them into *k *clusters and obtain $W_{kb}^{*},b=1,2,...B,k=1,2,...K$. We use PAM [31] for clustering. Considering the mean dispersion $\bar{l}=\sum_{b}\log(W_{kb}^{*})/B$ across *B *= 20 reference datasets, and their standard deviation $sd_{k}=\sqrt{\sum_{b}{\left(\log(W_{kb}^{*})-\bar{l}\right)}^{2}/B}$, the item $s_{k}=sd_{k}\sqrt{1+B^{-1}}$ is taken into consideration for a better control about the rejection of null model [12], and estimated cluster number *k' *is:

(4) $$\begin{array}{l} Gap(k)=\frac{1}{B}\sum_{b}\log(W_{kb}^{*})-\log(W_{k})\\ diff(k)=Gap(k)-(Gap(k+1)-s_{k+1})\\ k^{'}=\underset{k=1,2,...K}{\inf}(diff(k)\geq 0) \end{array}$$

The reference distribution is a null model of data structure. In [12], reference datasets are sampled uniformly either from the range of observed values for each feature, or the range of a box aligned with the principle components of data. However, it is encouraged to estimate reference distribution from existing samples rather than simply using uniform distribution because the bounding box of the whole dataset always includes some "blank" area. The existing cluster definition can help us focus on where the data really lies, and avoid generating a reference dataset violating the properties of original one. Here, we sampled $S_{m}^{'}$ from GMM of each existing phenotype and it makes sense to use this GMM as the reference distribution for $S_{m}^{'}$.

The problem now is to generate reference dataset from GMM, because $S_{m}^{'}$ is combined with new image E to form the dataset F, and the model of E is unavailable. We have to deal with $S_{m}^{'}$ and E separately because the distribution of E is unavailable. We propose to solve this problem by generating reference dataset from $S_{m}^{'}$ (GMM reference distribution) and E (uniform reference distribution) separately and combine two sets together, i.e. substituting $Supp(F)=Supp(E\cup S_{m}^{'})$ with

(5) $$P\_Supp(F)=Supp(E)\cup Supp(S_{m}^{'})$$

We discuss more detail of this strategy in the [Additional file 1], and supply a figure as [Additional file 6] to illustrate the motivation and innovation of our strategy.

Gap statistics method repeatedly carries out clustering using a set of candidate cluster numbers, and pick up the number supplying best within cluster dispersion as estimated cluster number. GMM is an accurate model for $S_{m}^{'}$, and using GMM as reference distribution can avoid the risk of split biological meaningful clusters and retain biological properties of existing phenotypes. The selection of *K *and *B *controls the number of clustering operation to be carried out and greatly influences the complexity of the whole methods. An effective way of utilizing existing phenotypes can greatly reduce *K *and make gap statistics method more suitable for online phenotype discovery in high-throughput image-based screens.

### Cluster definition and merging

We use Partitioning Around Medoids (PAM; also known as K-medoids) [31] to do clustering on combined set F. PAM provides better flexibility and robustness of choosing suitable dissimilarity measurements for different applications [32] and more efficient compared to Fuzzy clustering methods, especially in our cases where clustering are carried out frequently.

After clustering, we get a non-overlapping partition of $F=\overset{K^{'}}{\underset{m=1}{\cup }}F_{m},\forall m,n\in \left\{1,2...K^{'}\right\},F_{m}\cap F_{n}=\emptyset$, where the cluster number *K' *is determined through gap statistics, and we adjust the cluster labels to make sure that F_1 _includes the largest ratio of samples from existing phenotype $S_{m}^{'}$. Merging operation is done according to this partition. Datasets $S_{m}^{'}$, *m *∈ {1, 2,... *K*_0_} are combined with E one by one, and in each loop, the overlapped part of $S_{m}^{'}$ and E (if any) is located in F_1_, deleted from E and included as part of existing cluster:

(6) $$\begin{array}{l} E\leftarrow E-(F_{1}\cap E)\\ S_{m}\leftarrow S_{m}\cup (F_{1}\cap E) \end{array}$$

after all merging loops, clusters left in E are defined as new phenotypes.

The above merging strategy is based on theoretical case of $F_{1}⊇S_{m}^{'}$, but in reality, when we consider random sample set $S_{m}^{'}$ from GMM of an existing phenotype, it is possible that some samples are randomly far from the centre of existing phenotype and thus assigned into different clusters with their majority counterparts in $S_{m}^{'}$. We take two strategies to protect the merging operation from influence of outliers.

(1) Merging operations only happens when some cells in E are assigned into F_1_, together with more than 95% of samples $S_{m}^{'}$. And such cells are considered candidates for merging operation.

(2) For each merging candidate in E, we carry out statistical test with Bonferroni correction. We calculate the *p *value for each candidate with respect to the GMM for $S_{m}^{'}$, i.e. possibility of obtaining a value at least as extreme as (if not more) this candidate under the GMM. The corrected *p *value for each candidate is defined as its *p *value with respect to the GMM of $S_{m}^{'}$ divided by number of existing phenotypes *K*_0_. If the corrected *p *value is lower than 0.05/*K*_0_, the merging operation is rejected and we keep that candidate in E.

In the merging loops, we focused on identifying samples of existing phenotypes from E. While some cells of E are merged into existing phenotypes, novel clusters gradually stand out.

## Authors' contributions

NP and CB conceived the biological hypothesis while STCW and XZ conceived the bioinformatics part of the study. ZY and XZ designed the framework of online phenotype discovery and performed tests, CB and colleagues carried out genome-scale RNAi screens, helped to set up initial phenotype database and validated final results, FL developed the image processing methods. All authors contribute in writing this paper, have read and approved the final version of this manuscript.

## Supplementary Material

Additional file 1 **Supplementary materials on performance validation and algorithm details**. Methods of simulating real cells using polygons are noted. Documents and results for two more validation experiments are provided, and two published dataset using different organisms were involved in these experiments. More details of four existing phenotypes used in the main text are presented, including the histogram of specific feature and key parameters for estimated GMM. Our improvement on gap statistics method and one-class SVM novelty detection are also discussed in detail. Click here for file

Additional file 2 **Performance comparison on restoring biological meaningful cluster from published high throughput screen dataset**. These two histograms report the comparisons on the ability of restoring biological meaningful pheno-clusters between our method and SVM based method. The comparison carried out on a published high throughput screen dataset based on *Drosophila *BG-2 cell line, and results using two different groups of existing phenotypes are presented separately. Click here for file

Additional file 3 **Typical images and information for datasets of four cell cycle phases in HeLa cells**. In this figure, typical images and some information from a published dataset of HeLa cells are summarized. This dataset consists of single channel fluorescent images of HeLa nuclei in four cell cycle phases and it was used to illustrate the prospect of combining our method to dataset from various organisms. Click here for file

Additional file 4 **Performance comparison on cell cycle phase identification using HeLa dataset**. These three histograms report performance comparisons between our method and SVM based method. The comparisons were carried out on the HeLa dataset described in Additional file 3, and the results using three different groups of existing phenotypes are presented separately. Click here for file

Additional file 5 **Information on four existing phenotypes for case 1–4: histogram for major axis length and complete model parameters**. This figure extends the information in Figure 7 of main text. The histogram for major axis length helps to show the necessity of modelling each morphological feature using GMM, and the parameters of estimated models are also available. Click here for file

Additional file 6 **Improving the strategy of taking reference dataset for gap statistics: motivation and innovation**. This figure illustrates why we have to modify the strategy of taking reference dataset in the context of online phenotype discovery and how we work it out. The limitations of existing method, as well as the idea of our improvement are illustrated. Click here for file
