# Supplementary material for: Using iterative cluster merging with improved gap statistics to perform online phenotype discovery in the context of high-throughput RNAi screens
Source: BMC Bioinformatics. 2008 Jun 5;9:264. doi: 10.1186/1471-2105-9-264 (PMC2443381; doi:10.1186/1471-2105-9-264)
Supplement: Additional file 1 — Supplementary materials on performance validation and algorithm details. Methods of simulating real cells using polygons are noted. Documents and results for two more validation experiments are provided, and two published dataset using different organisms were involved in these experiments. More details of four existing phenotypes used in the main text are presented, including the histogram of specific feature and key parameters for estimated GMM. Our improvement on gap statistics method and one-class SVM novelty detection are also discussed in detail. [file 1471-2105-9-264-S1.doc]

# Supplementary materials on performance validation and algorithm details

# Using iterative cluster merging with improved gap statistics to perform online phenotype discovery in the context of high-throughput RNAi screens

### Zheng Yin^1, 3^*, Xiaobo Zhou^1^*, Chris Bakal^2^*, Fuhai Li^1^, Youxian Sun^3^, Norbert Perrimon^2^, Stephen TC Wong^1§^

^1^Center for Bioinformatics, The Methodist Hospital Research Institute and Weill Cornell College of Medicine, 6565 Fannin Street, Houston, TX, 77030, USA.

^2^Department of Genetics and Howard Hughes Medical Institute, Harvard Medical School, 77 Avenue Louis Pasteur, Boston, MA, 02115, USA.

^3^State Key Laboratory of Industrial Control Technology, Zhejiang University, 38 Zheda Road, Hangzhou, Zhejiang Province, 310027, China.

*These authors contributed equally to this work

^§^Corresponding author

## Simulating real cells using seven types of polygons

From fluorescent microscopy cell images generated for *Drosophila* genome wide RNAi screen, we picked up seven types of cells (RGB colour image) and simulated them using seven types of polygons. All simulated polygons were illustrated using 8-bit gray level images (256 level gray scale with intensity 0 indicating black and 255 representing pure white).

The key geometry and gray level parameters of each polygon are sampled from a series of random variables, which are defined according to the information summarized from real cells. For instance, we sampled from normal distribution with zero mean and standard deviation of 0.3, took absolute value of the samples and used them as the eccentricity for ellipses and rings; gray levels filled into an ellipse are sampled from uniform distribution in the range of [0, 50], while the rectangles are filled with gray levels sampled uniformly from the range of [120, 180]. Each polygon is described using six features, including mean and standard deviation of gray levels, and a group of geometry features, namely length of longest axis, length of shortest axis, perimeter and area of the polygons.

## Performance validation on published genetic screening dataset aiming at defining local signalling networks regulating cell morphology

3a

3b

### Dataset description

We downloaded quantified data related to high throughput RNAi screen in [s1] from [s2] and use it to validate our method’s ability of restoring biological meaningful clusters. This dataset quantifies the morphological change of 12601 cell segments from 249 different treatment conditions (TCs). In each TC, *Drosophila* genes are either systematically over-expressed and/or inhibited by the use double-stranded RNA (dsRNA). A quantitative morphological signature (QMS) is defined for each TC based on the morphological change it causes. All TCs are clustered based on their QMS, and seven out of 41 resulted clusters are highlighted in [s1]. We selected altogether 6782 cell segments from six of these biological meaningful clusters and use their quantified morphology indicators to carry out our experiments. The information of those six clusters is summarized in Table S1, and more information related to the work in [s1] can be found in supplementary materials.

The work in [s1] represents the recent attempt of using fluorescent microscopy images based genetic screen to define local network regulating cell morphology. A QMS is defined for each of the 249 TCs according to the observed morphological change on cultured *Drosophila* BG-2 Cells. Three levels of quantification are employed to generate a QMS for each TC: each individual cell image/segment is first quantified by 145 morphological features; the similarity of all cells in the dataset to twelve “reference phenotypes” is measured using twelve neural networks (NN) classifiers that distinguish training /reference phenotypes from each other; and finally a normalized Z-score for all cells from the same TC. Seven of twelve scores are used to form final QMS vector for each TC.

### Restoring biological meaningful clusters

In this case we try to validate the ability of restoring biological meaningful phenotypes using our method. Six clusters highlighted in [s1], denoted as Cluster 6, 8, 18, 33, 1 and 27 were used. Quantified morphological indicators for altogether 2,800 cell segments were divided into a flow of image input consisting of 28 synthetic images with 100 cells in each image. Two experiments with different sets of existing phenotypes were carried out and the performance of our method was compared with four SVM based method with different parameters. Same as the experiments in main text, the accuracy with respect to each cluster/ phenotype was defined as the proportion of test samples restored into its original cluster. Both experiments (using our method and SVM based method, respectively) were repeated 100 times with different order of inputting test images.

The average accuracies of both experiments are shown in [Additional file 2]. In general, the trend of performance is similar with those for synthetic datasets, with our method outperforming all SVM based method in accuracies for at least three of six clusters. When four clusters (6, 8, 18 and 33) serve as existing phenotypes [Additional file 2, *left*], we obtain more stable performance with smaller standard deviation across different clusters. On the other hand, when cluster 33 is removed from the set of existing phenotypes [Additional file 2, *right*], we get higher accuracy on cluster 8 and 18 as well as degraded performance for other clusters, especially cluster 27. Cluster 18 is the largest pheno-cluster in the dataset, featuring large, flat cells typically with extensive lamellipodia, and cluster 27 includes three TCs featuring cells displaying an aberrant number of long protrusions [s1], by adding cluster 33 (featuring cells subject to dsRNA TCs targeting at *Rho1* family) to existing phenotypes, we can identify cells with spiky and polarity structure better, thus reduce the possibility of merging cells in cluster 27 into cluster 18.

This case shows our method’s ability of restoring biologically meaningful clusters/ phenotypes in the online scenario, and hence, can be used to extend genetic screen in [s1] to genome wide scale. In the supplementary of [s1], the authors raise the question about “indistinguishable phenotypes” in cluster 31, where treatment conditions related to three distinct phenotypes (wild type, *CG3799* over-expression and *Rac14V* over-expression), which are all with prolonged shape but different polarity properties, can not be distinguished. One of the reason for such problem is that those undistinguishable TCs actually include cells with distinct phenotypes, and the ratio of cells in those different phenotypes are comparable to each other, but the scores for all those cells are normalized and averaged to form the signature for each TC. Therefore, the information included in multiple phenotypes is diminished.

## Applying the proposed method to cell phase identification of HeLa cells

We validated the proposed online phenotype discovery method using the dataset related to cell cycle phase identification of HeLa cells in automated microscopy [s3], and the result indicates the prospect of using our methods on high-throughput dataset from various organisms.

### Cell culture and image acquisition

The data is generated following the culturing protocol described in [s3]. In brief, HeLa H2B-GFP cells were thawed 6 days and cultured in DMEM with 10% FBS. Cells were incubated at 37°C in 5% CO_2_. All cells were plated in 8 well #1 German borosilicate sterile bottomed plates (Nalge Nunc International) 18h before imaging at 25,000 cells per well (50, 000 cells per ml). Images were acquired 15 minutes apart during a 50h period on an automated epi-fluorescence TE2000-E Eclipse microscope (Nikon Instruments Inc., USA) with a motorized XYZ-plane stage. A total of 200 images for each position were acquired and exported as 16bit uncompressed TIFF files. More details of cell culture and image acquisition are available in [s3].

**Cell segmentation, feature extraction and feature selection**

The obtained images have one channel and focus on the information of cell nuclei. Herein we adopted the validated cell segmentation method proposed in [s3]: cell shape information is first obtained with a binarization process, then both intensity and shape information is used for local maxima generation and next gradient vector field are utilized to locate center of cells (local maxima), finally the detected cells are segmented via a seeded watershed algorithm.

Same as the newer *Drosophila* high-throughput dataset utilized in the main text of our paper, a same set of 211 morphology features from five categories are defined to describe each segmented cell. The difference is that in this HeLa dataset all the features are extracted from images with only one channel. Regarding to the analysis of similar dataset, and the necessity of specific job, different strategies have been proposed to carry out feature reduction [s3, s4]. Considering our job of identifying novel phenotypes from different groups of existing phenotypes, we continue to use the unsupervised feature selection method proposed in [s5]. An informative subset of sixteen features is selected. The geometric feature of “perimeter” is selected, along with 6 Gabor features, 4 moment features, 2 texture features and 3 shape features.

**Experiments and results**

We selected altogether 2753 images of segmented HeLa cells, and carried out expert ground truth labeling to classify them into four cell cycle phases, namely Inter-phase, Prophase, Metaphase and Anaphase. Typical images and number of cells in each phase for this selected dataset are presented in [Additional file 3]. Two rows of typical images for anaphase indicate one challenge of identifying cell cycle phases: the polymorphism of images related to a same phase. In the upper row, two separated chromosomes can be clear identified in opposite end of the mitotic spindle, but still remain in the same segment. Such images follow the definition of anaphase, however it is difficult to acquire such images using our protocol, thus more images of anaphase (up to 85%) have cells showing just separated chromosomes, i.e. cells with small size, prolonged shape and high intensity, like shown in the lower row of typical images for anaphase. These two types of anaphase images differ from each other greatly in size, shape and texture, thus add to the difficulty of automatic identification. In such occasion, our strategy of modelling existing phenotypes using mixture models is suitable to retain biological meaningful clusters.

We tested our method using a series of cross validation. In each experiment, two or three cell cycle phases were used as “existing phenotypes”, while the others were considered as “novel”. The dataset of existing phenotypes were divided evenly into five parts, and the GMM of existing phenotypes were estimated using 80% of the dataset, while the other 20% cells of existing phenotypes were combined with cells from the “novel” phenotype as test dataset. The test dataset were divided into small groups with 80-100 cells each to simulate a series of image input. We tested our methods under six different combinations of existing phenotypes, for each combination the five-fold cross validation were carried out 100 times, and the average accuracy on each cell phase with standard deviation across all the experiments are reported in Table S2 with the definitions of accuracy for existing and novel phenotypes remain the same as in the main text. It can be seen that when anaphase served as existing phenotype, the two subtypes of images can be effectively modelled by GMM and the test samples of different styles can be accurately restored into their original phenotype. The accuracy for anaphase slipped when it served as novel phenotype, but the never fell below 80%.

[Additional file 4] compares the average accuracy obtained by our method and SVM methods with different parameters, the results from three different combinations of existing phenotypes are recorded. Our method out-performed SVM based method in almost every experiment, in one rare exception of [Additional file 4 *right*], SVM with nu=0.1 slightly outperformed our method on anaphase (86.2% vs 85.7%), however its accuracies for prophase and metaphase were below 75%. Our method avoided the imbalance caused by SVM with different parameters, and performed consistently in the identification of cell cycle phases on HeLa cell dataset.

## Discussion on using GMM for phenotype modelling

Instead of hanging with single Gaussian model, we are using Gaussian mixture model to describe each biological meaningful cluster. Theoretically GMM can approximate closely any continuous density function for a sufficient number of mixtures and appropriate model parameters [s6]. In this study, we checked the histogram of the samples under different phenotypes. We found Gaussian mixture model can be applied to model the distribution. Considering the issue of space, we deleted in the first version. In the revision, we present such information in [Additional file 5].

[Additional file 5] shows the information of feature distribution for the four existing phenotypes used in Case 1-4 in the original paper. In column three of [Additional file 5], a histogram is shown for each phenotype, and it shows the distribution of Major axis length, one of the selected features to describe each cell, and the parameter for this feature in the final GMM is also shown. In column four, the complete GMM estimated for each phenotype is presented.

## Improving the strategy of sampling reference dataset in gap statistics method

We improved the strategy of defining reference distribution and sampling reference dataset for Gap Statistics method [s7]. The reference distribution is a null model of data structure. In [s7], reference sets are sampled uniformly either from the range of observed values for each feature, or the range of a box aligned with the principle components of data. As shown in the left part of [Additional file 6]. This brings a problem that, when the dataset contains distinct clusters, there would be blank area (indicated using blue circles) in the support defined by bounding boxes align with the whole dataset. Although the bounding box aligned with the principle component can partly solve this issue, the blank area remains (indicated by blue rectangle). Worse still, the bounding boxes define the support of the reference distribution, the reference dataset are sampled from such distribution while the sample number is usually selected as the same as the real dataset [s7]. Thus, we have a “sparser” dataset than the real one, because some samples are from the blank area.

On the other hand, we define the reference distribution to supply a null hypothesis of the data structure reflecting the situation when the dataset was mono-genous. Now that we have built GMM for existing dataset, and using uniform distribution as null hypothesis would bring the risk of splitting biological meaningful clusters. It is also encouraged in [s7] that the information of existing phenotype be included because uniform distribution is not necessarily the optimal choice of reference model when the feature space dimensionality is bigger than one.

By taking reference distribution from separate binding boxes, we shrink the blank area caused by the difference between existing clusters, thus the reference dataset can focus on where data really lies. By using the GMM as the reference distribution, we bring in the exact information on the mono-genous dataset, thus we can avoid splitting existing phenotypes, even if they are formed by quite distinct Gaussian items. The right part of [Additional file 6] illustrates the innovation of our methods.

## Novelty detection and one-class SVM

Novelty detection problem is formulated as follows: given a set of independently identically distributed (i.i.d.) training samples,$x_{1},...,x_{n}\in X\subseteq\mathbf{R}^{N}$, drawn from a probability distribution in feature space, ***P***, the goal of novelty detection is to determine the ‘simplest’ subset, ***S***, of the feature space such that the probability that an unseen test point, $x^{'}$, drawn from ***P*** lies outside of ***S*** is bounded by an a priori specified value, $\nu\in(0,1]$. In other words, such problem is handled as a simplified version of density estimation problem. One-class SVM, introduced in [s8], is widely used in novelty detection. In the formulation of one-class SVM, existing dataset are first mapped into a feature space using a kernel function $K(x,y)=(\Phi(x)\cdot\Phi(y))$and then maximally separated from the origin using a hyper-plane , a decision function is thus obtained for each unseen test point $x^{'}$,$f(x^{'})=\mathrm{sgn}(w\cdot\Phi(x^{'})-\rho)$ , specific label, e.g. -1 is defined for outliers and the parameters$\{w,\rho\}$ are determined by solving a quadratic programming problem similar to the basic SVM case. Compared with basic SVM, a parameter $\nu\in(0,1]$ is involved in this problem, and it is the asymptotic upper bound of training data which are labelled as outliers. Both training and test samples are classified into two categories: known and novel. If the training set itself is not homogenous, the set of support vector would vary dramatically with $\nu$, so $\nu$ should be selected according to the prior knowledge of outlier existence. We use a Gaussian kernel with width of 0.5 to map the features into high dimension space, i.e. $K(x,y)=e^{-0.5*\left\|x-y\right\|^{2}}$

## Modifying one-class SVM based novelty detection to fit it into the context of online phenotype discovery

Novelty detection methods do not automatically fit in the scenario of online phenotype discovery. These methods need to continuously re-train their model so that the newly discovered phenotypes won’t be considered as outliers in the future. However, such re-training is intractable when millions of existing cells pile up and new images are continuously generated. Worse still, novelty detection cannot differentiate multiple existing and novel phenotypes.

We modify one-class SVM based novelty detection into a two-step method for each new image and compare its performance with our method. In the first step, we carry out traditional novelty detection to new images, but we use the huge existing dataset only once while iteratively updating the support vectors of one class model with new images. After novelty detection with one image, we re-train the one-class model, only keep the support vectors and combine these support vectors with the next new image. In the second step, we train a series of linear SVM with samples from one pair of existing phenotypes, and classify each sample in new image using multiple SVM and determine its phenotype by majority vote among all SVM.

We propose a possible way to extend novelty detection method in the scenario of online phenotype discovery with multiple existing and novel phenotypes. Such methods can be improved through more careful selection and training of classifiers and refined design of model updating procedures.

# Reference

s1. Bakal C, Aach J, Church G, Perrimon N: **Quantitative morphological signatures define local signalling networks regulating cell morphology.** *Science* 2007, **316**, 1753-1756

s2. Supporting online materials: quantitative morphological signatures [<http://arep.med.harvard.edu/QMS>]

s3. Wang M, Zhou X, Li FH, Huckins J, King RW, Wong STC: **Novel cell segmentation and online SVM for cell cycle phase identification in automated microscopy.** *Bioinformatics* 2008, **24**(1), 94-101

s4. Wang M, Zhou X, Li FH, King RW, Wong STC: **Novel cell segmentation and online SVM for cell cycle phase identification in automated microscopy.** *BMC* *Bioinformatics* 2007, **8**(32)

s5. Mitra P, Murthy C A, Pal S: **Unsupervised feature selection using feature similarity,** *IEEE Transactions on Pattern Analysis and Machine Intelligence* 2002, **24**(3), 301-312

s6. Zhao Y, Zhuang X, Ting S: **Gaussian mixture density modeling of non-Gaussian source for autoregressive process.** *IEEE Trans. Signal Processing* 1995, **43** (4), 894-903.

s7. Tibshirani R, Walther G, Hastie T: **Estimating the number of clusters in a dataset via the gap statistic,** *Journal of Royal Statistics Society*. B 2001 **32**(2), 411-423

s8. Schölkopf B, Platt J, Shawe-Taylor J, Smola A, Williamson R: **Estimating the support of a high dimensional distribution.** *Neural Computation* 2001, **13**, 1443-1471

# Supplementary figure legends

## Additional file 2-Performance comparison on restoring biological meaningful cluster from published high throughput screen data

Given certain group of existing pheno-clusters, and images including multiple (novel and existing) phenotypes, the accuracy values for six phenotypes across 100 image input orders are shown. Four different sets of parameters are used for SVM based method. *Left* Cluster 6, 8, 18 and 33 are existing phenotypes; *Right* Cluster 6, 8 and 18 are existing phenotypes.

## Additional file 3-Typical images and information for datasets of four cell cycle phases in HeLa cells

## Additional file 4-Performance comparison on cell cycle phases identification using HeLa dataset

Given certain group of existing phenotypes, and images including multiple (novel and existing) phenotypes, the accuracy values for four cell cycle phase across 100 times five-fold cross validations are shown. Four different sets of parameters are used for SVM based method. *Left* Dataset for inter-phase and prophase are existing phenotypes; *Middle* Dataset for inter-phase and metaphase are existing phenotypes; *Right* Dataset for inter-phase and anaphase are existing phenotypes.

## Additional file 5-Information on four existing phenotypes for case 1-4: histogram for major axis length and complete model parameters

## Additional file 6-Improving the strategy of taking reference dataset for gap statistics: motivation and innovation

# Supplementary Tables

## Table S1. Information for six clusters selected from quantified dataset in [s1]

| *Cluster # in [s1]* | *6* | *8* | *18* | *33* | *1* | *27* |
| --- | --- | --- | --- | --- | --- | --- |
| Annotation for  clusters in [s1] | Protrusion/ Adhension formation | Lamellipodia formation | Adhension disassembly/  cortical tension | Rho1  Cluster | Rac1  cluster | MT  capture |
| # of TCs | 37 | 25 | 74 | 19 | 4 | 3 |
| # of cells for training | 1183 | 853 | 1039 | 877 | 0 | 0 |
| # of cells for testing | 1000 | 300 | 600 | 200 | 400 | 300 |
| Total # of cell segments | 2183 | 1153 | 1639 | 1077 | 422 | 308 |

## Table S2. The performance of our method in cross validation on cell cycle phase dataset

| Mitosis phases | Avg. accuracy (%) with (standard deviation, %) across 100 times five-fold cross validation using different experiment design  (results for novel phenotypes are labeled using ***italic and bold font***) | | | | | |
| --- | --- | --- | --- | --- | --- | --- |
| Inter-phase | 89.2 (2.2) | 90.4 (1.6) | 91.4 (2.1) | 87.3 (2.7) | 86.7 (1.9) | 86.4 (2.2) |
| Prophase | ***82.1 (3.1)*** | 88.1 (2.9) | 87.6 (3.4) | 84.9 (2.8) | ***81.6 (2.9)*** | ***82.7 (3.1)*** |
| Metaphase | 86.4 (2.6) | ***85.7 (2.8)*** | 88.7 (3.4) | ***83.8 (2.6)*** | 85.2 (2.1) | ***84.1 (2.5)*** |
| Anaphase | 90.6 (1.9) | 89.7 (2.2) | ***83.4 (3.4)*** | ***84.6 (3.1)*** | ***84.9 (2.9)*** | 85.7 (2.4) |
