# Supplementary figures and images for: Using iterative cluster merging with improved gap statistics to perform online phenotype discovery in the context of high-throughput RNAi screens
Source: BMC Bioinformatics. 2008 Jun 5;9:264. doi: 10.1186/1471-2105-9-264 (PMC2443381; doi:10.1186/1471-2105-9-264)

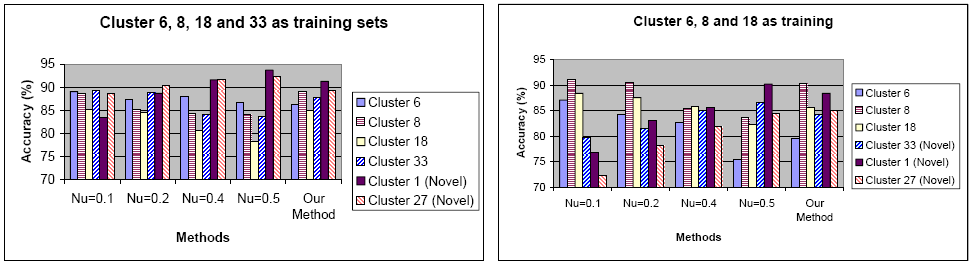

Supplement: Additional file 2 — Performance comparison on restoring biological meaningful cluster from published high throughput screen dataset. These two histograms report the comparisons on the ability of restoring biological meaningful pheno-clusters between our method and SVM based method. The comparison carried out on a published high throughput screen dataset based on Drosophila BG-2 cell line, and results using two different groups of existing phenotypes are presented separately. [file 1471-2105-9-264-S2.tiff]

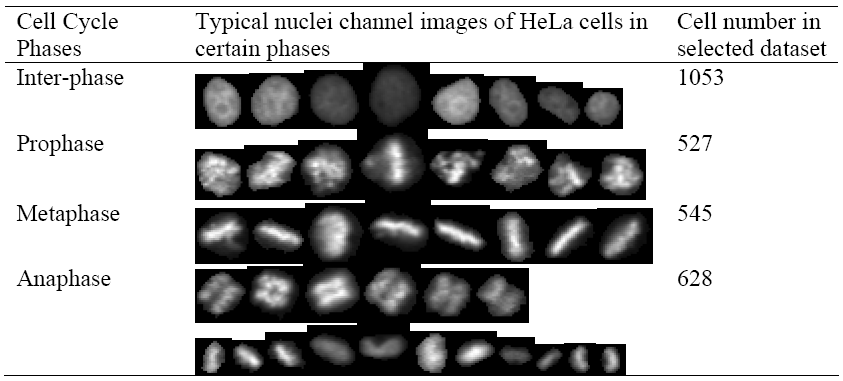

Supplement: Additional file 3 — Typical images and information for datasets of four cell cycle phases in HeLa cells. In this figure, typical images and some information from a published dataset of HeLa cells are summarized. This dataset consists of single channel fluorescent images of HeLa nuclei in four cell cycle phases and it was used to illustrate the prospect of combining our method to dataset from various organisms. [file 1471-2105-9-264-S3.tiff]

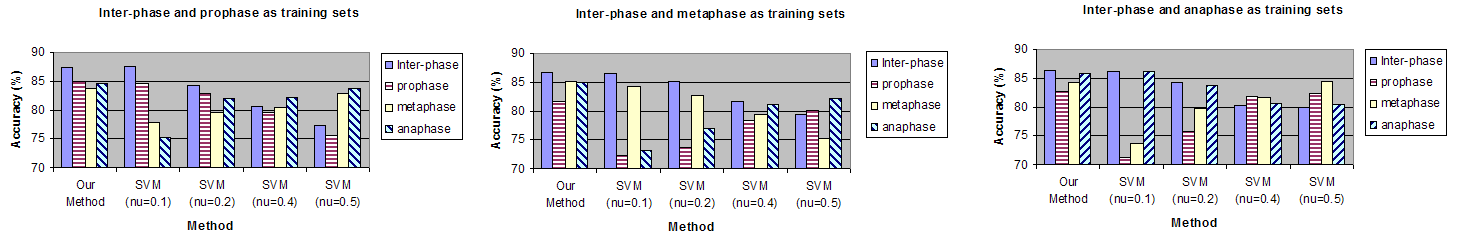

Supplement: Additional file 4 — Performance comparison on cell cycle phase identification using HeLa dataset. These three histograms report performance comparisons between our method and SVM based method. The comparisons were carried out on the HeLa dataset described in Additional file 3, and the results using three different groups of existing phenotypes are presented separately. [file 1471-2105-9-264-S4.tiff]

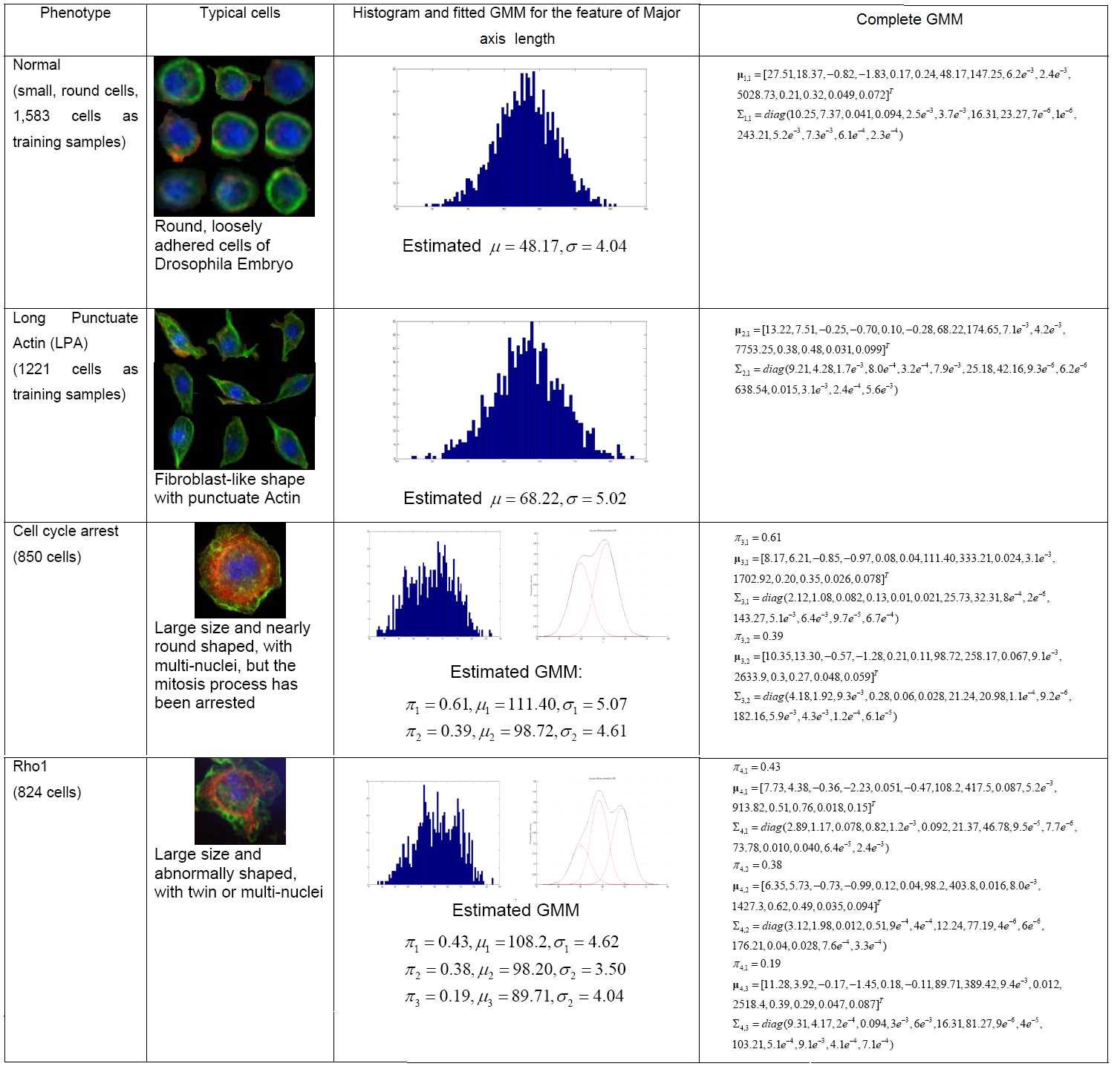

Supplement: Additional file 5 — Information on four existing phenotypes for case 1–4: histogram for major axis length and complete model parameters. This figure extends the information in Figure 7 of main text. The histogram for major axis length helps to show the necessity of modelling each morphological feature using GMM, and the parameters of estimated models are also available. [file 1471-2105-9-264-S5.tiff]

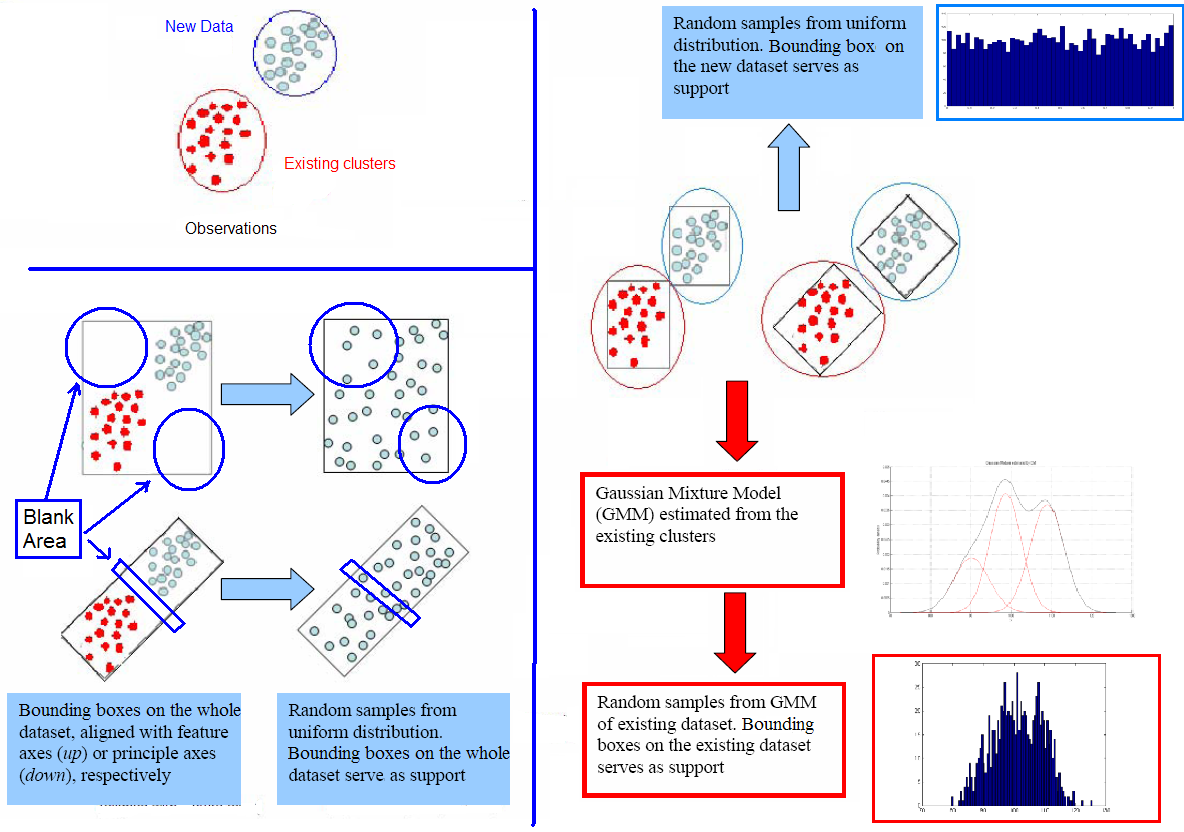

Supplement: Additional file 6 — Improving the strategy of taking reference dataset for gap statistics: motivation and innovation. This figure illustrates why we have to modify the strategy of taking reference dataset in the context of online phenotype discovery and how we work it out. The limitations of existing method, as well as the idea of our improvement are illustrated. [file 1471-2105-9-264-S6.tiff]
